# Emotional modulation of inhibitory control in rumination from empirical and computational perspectives

**DOI:** 10.3758/s13415-025-01360-7

**Published:** 2025-10-31

**Authors:** Selena Singh, Benjamin Li, Serenna Gerhard, Abraham Nunes, Suzanna Becker

**Affiliations:** 1 Department of Psychology, Neuroscience and Behaviour, McMaster University, Psychology Building, 1280 Main Street West, Hamilton, ON L8S 4L8 Canada; 2 Sunnybrook Research Institute, 2075 Bayview Ave, North York, ON M4N 3M5 Canada; 3 Department of Psychiatry and Faculty of Computer Science, Dalhousie University, 3083B-5909 Veterans Memorial Lane, Halifax, NS B3H 2E2 Canada

**Keywords:** Rumination, Brooding, Inhibitory control, Emotion, Computational modeling

## Abstract

Rumination involves repetitive dwelling on negative thoughts, emotions, and memories and is a risk factor for depression. Cognitive theories suggest that rumination stems from heightened automatic, emotional stimuli-driven (i.e., “bottom-up”) and/or deficits in effortful, goal-directed (i.e., “top-down”) processes. It remains unclear whether rumination arises from bottom-up processes impacting top-down inhibitory control or from impaired inhibition alone. We used both experimental and computational approaches to address this. Participants (*N* = 151) first completed self-report measures of trait rumination, followed by the standard and emotional Stroop tasks, before and after a rumination induction. Brooding, a maladaptive component of rumination, was associated with slower reaction times for both tasks. A rumination induction, expected to heighten bottom-up emotional salience, increased the congruency effect in proportion to brooding severity. To study underlying computational mechanisms, we adapted an existing parallel distributed processing model of the Stroop task to include mechanisms for emotional cue processing and subsequently numerically fit the model parameters to individual participant Stroop data. Brooding was positively associated with bottom-up weights and steeper neural activation curves in the task control layer, representing a greater sensitivity to emotional cues and changes in task demands. Higher brooding also predicted faster temporal integration (i.e., activity decay) of top-down control signals and slower temporal integration (i.e., activity persistence) of emotional cues. We therefore propose that a greater sensitivity to changes in task demands and bottom-up emotional cues, along with a diminished capacity to sustain goal-relevant control signals, underlie inhibitory control deficits in trait rumination.

**Supplementary Information:**

The online version contains supplementary material available at 10.3758/s13415-025-01360-7.

## Introduction

Rumination involves repetitive dwelling on thoughts, moods, and past events. It can include both negative and positive thought content, with the negative form highly relevant to psychopathology. As a transdiagnostic symptom of mood-, anxiety-, personality-, sleep-, and stress-related disorders (Baer et al., 2012; Cox et al., 2019; McLaughlin & Nolen-Hoeksema, 2011; Moulds & McEvoy, 2025; Ruscio et al., 2011; Spinhoven et al., 2015), rumination plays a substantial role in the onset and persistence of negative affect (Fresco et al., 2002; Kirkegaard Thomsen, 2006; Thomsen et al., 2003). A tendency to ruminate may remain stable despite changes in levels of negative affect, suggesting that rumination may be a trait-like phenomenon. High levels of trait rumination is associated with the worsening of depressive symptoms and predicts the duration and onset of major depressive episodes (Just & Alloy, 1997; Nolen-Hoeksema, 2000; Nolen-Hoeksema et al., 2008). Ruminative worry as an anxiety response may also drive depression-anxiety comorbidity by reinforcing feelings of hopelessness and maladaptive perfectionism (Harris et al., 2008). It is therefore clinically valuable to understand the underlying neurocognitive mechanisms of rumination, which may inform treatments for various psychopathologies.

Depressive rumination is a form of repetitive negative thinking, an umbrella term that encompassess self-focused, abstract, and perseverative negative thought processes (Ehring & Watkins, 2008; Ikani, 2021; Moulds & McEvoy, 2025). Repetitive negative thinking includes both anxious worry and depressive rumination, which are often correlated (Hong, 2007; Stade & Ruscio, 2023), thought to share underlying mechanisms, and distinguished primarily by thought *content*: worry centers on threat-related processes (e.g., “I’m going to embarrass myself”), whereas rumination is more closely tied to self-deprecation and negative mood (e.g., “I’m a failure”) (Ehring & Watkins, 2008; Moulds & McEvoy, 2025; Wahl et al., 2019). In depression specifically, rumination can be subdivided into brooding and reflective pondering. Brooding, considered the maladaptive subtype, predicts concurrent and future depressive symptom severity, whereas reflective pondering predicts *lower* future depressive symptoms (Treynor et al., 2003). This two-factor model is further supported by findings that brooding and reflective pondering differentially relate to cognitive biases in depression, with brooding associated with negative attentional biases independently of other depressive symptoms (Joormann et al., 2006). Different components of rumination thus appear to have distinct relationships with depressive symptomology, reflecting both potentially adaptive and maladaptive processes. The present study focuses on the maladaptive form: ruminative brooding. Nevertheless, it is important to consider that forms of repetitive negative thinking may share underlying mechanisms that contribute to the perseverative nature of these thought patterns (Ehring & Watkins, 2008; Moulds & McEvoy, 2025) and are therefore highly relevant even when studying brooding specifically.

Cognitive theories of rumination can be generally classified as “bottom-up” and/or “top-down.” Here, “bottom-up” and “top-down” are terms akin to “hot” and “cold” cognition, referring to emotion-dependent and emotion-independent cognition, respectively (Roiser & Sahakian, 2013). Top-down cognition is goal-directed and effortful (Kveraga et al., 2007; Posner & Petersen, 1990; Sarter et al., 2001), governed by the prefrontal cortex (Ochsner et al., 2009), and regulates automatic, stimulus-driven bottom-up processes (Buhle et al., 2014; Kohn et al., 2014), generated by limbic circuitry (Etkin et al., 2015; MacLean, 1949; Murphy et al., 2003). Stimuli relevant to bottom-up processes include both emotionally salient environmental and endogenous (i.e., internally generated) stimuli given that the limbic system is involved in the detection and regulation of both of these types of cues. Dysregulation within top-down and bottom-up circuits may contribute to cognitive impairments and emotional pathology, driven by reduced prefrontal top-down control and/or excessive limbic bottom-up activation (Buhle et al., 2014; Johnstone et al., 2007; Kovner et al., 2019; Morawetz et al., 2017; Ochsner et al., 2004). These pathologies include major depression (Johnstone et al., 2007), bipolar disorder (Lagopoulos & Malhi, 2011), and age-related cognitive impairment (Gazzaley et al., 2005; He et al., 2021). Below we review the top-down, bottom-up, and integrative theories of rumination.

Some bottom-up theories propose that both depressive rumination and anxious worrying are responses to heightened endogenous emotional stimuli (Borkovec et al., 2004; Davey, 2006; Mennin et al., 2005; Nolen-Hoeksema, 1991). Indeed, the first conceptualization of depressive rumination, from the *response style* hypothesis, proposes that rumination is related to the habitual tendency to respond to negative moods, thoughts, and stressors by repetitively evaluating the causes, meanings and implications of them (Nolen-Hoeksema, 1991, 2000). This theory proposes that rumination functions to aid with emotional regulation. Indeed, subsequent theories have proposed that worrying in anxiety disorders arises due to a fear of emotion, and may serve as a mechanism for avoiding intense negative emotions in the short-term (Borkovec et al., 2004; Mennin et al., 2005); a similar process of cognitive and affective avoidance may also occur in depressive rumination (Krieger et al., 2013; Martell et al., 2001; Moulds et al., 2007; Watkins & Moulds, 2007). Others have proposed that rumination is a response to negative mood following a failed goal, which persists until positive mood is established (Davey, 2006). These theories each suggest that negative emotions, i.e., bottom-up endogenous cues, are the general origin of rumination.

These bottom-up theories, however, are mechanistically imprecise; they cannot explain the perseverative nature of rumination. To this end, a second class of top-down theories have been proposed, linking rumination to deficits in executive functions with clearer mechanistic bases, including monitoring and updating working memory, inhibition, and mental set shifting (Miyake et al., 2000). Rumination has indeed been related to inflexible working memory updating (Joormann et al., 2011; Meiran et al., 2011), with higher levels of trait rumination associated with greater difficulty removing irrelevant negative stimuli from working memory (Joormann & Gotlib, 2008, 2010; Zetsche et al., 2012). This inflexible working memory updating may be related to poor inhibitory control; participants with high trait rumination had difficulties disengaging from working memory material when instructed to switch attention to a different task (Whitmer & Banich, 2007). The *impaired disengagement hypothesis* therefore proposes that rumination (and brooding in particular) arises as a deficit in inhibitory control, where individuals may not be able to voluntarily disengage from their internally focused thoughts to engage with external stimuli (Koster et al., 2011). This theory has been supported by evidence from clinical research demonstrating that interventions that improve general attentional control mitigate rumination (Hammerdahl et al., 2025), and a meta analysis reporting significant negative associations between trait rumination and both inhibitory control and mental set shifting (Yang et al., 2017).

Although more mechanistically precise, top-down theories of rumination fail to account for the affective nature of rumination, identified as central to this thought process, and brooding in particular (McLaughlin & Nolen-Hoeksema, 2011; Nolen-Hoeksema, 1991; Nolen-Hoeksema et al., 2008). Integrative theories that explain bottom-up and top-down interactions may therefore be better suited for understanding rumination (Ikani, 2021). Attention and emotional processing have indeed been demonstrated to interact (Blair et al., 2007; Pessoa, 2008, 2013), possibly by virtue of bi-directional limbic and prefrontal neural circuitry (Arnsten & Rubia, 2012). Existing integrative theories of rumination include the resource allocation hypothesis and attentional scope model. The *resource allocation hypothesis* or *resource depletion account* (Hertel, 1998, 2004; Hoebeke et al., 2024; Levens et al., 2009; Philippot & Brutoux, 2008; Watkins, 2002) proposes that a pool of executive “resources” are shared between emotional processing and cognitive functions and that state rumination shifts executive resources away from cognitive functions, leading to deficits. This theory explains cognitive deficits during active rumination; however, it cannot explain mechanisms of *trait* rumination. Alternatively, the *attentional scope model* of rumination proposes that negative mood narrows “attentional scope,” or restricts the number of items in working memory to negative stimuli, leading to a ruminative state in trait ruminators (Whitmer & Gotlib, 2013). This theory proposes that individuals with a high tendency to ruminate will have an inherently narrow attentional scope that is further narrowed by negative mood, and the resulting constrained working memory resources will impair cognitive control. This theory is limited by assuming that attentional scope (independent of inhibition) is sufficient for explaining rumination. Inhibitory control facilitates attentional disengagement from task-irrelevant processes (O’Connor et al., 2012; e.g., rumination, Zetsche et al., 2012). Deficits in inhibitory control may therefore contribute to the enduring, perseverative quality of rumination. Furthermore, inhibition and attentional scope are functionally (Cowan et al., 2006) and neurobiologically distinct, governed by the frontal and parietal cortices, respectively (Li et al., 2017). Bottom-up influences on inhibitory control in trait rumination therefore should be investigated to construct an integrative theory centered around these processes.

The purpose of this study is to propose such an integrative theory of trait rumination, focusing on brooding specifically. To construct such a theory in a comprehensive manner, the impact of both types of bottom-up stimuli (i.e., endogeneous and exogeneous) on inhibitory control must be examined. The aims of this study are twofold: 1) investigate the impacts of bottom-up endogeneous and exogeneous emotional stimuli on inhibitory control in rumination, and 2) identify the underlying neural computational mechanisms. Identifying neural mechanisms via computational modeling forces components of a theory to be explicitly represented, directly addressing the mechanistic precision issues of previously proposed bottom-up theories. We hypothesize that rumination may be associated with baseline inhibitory control deficits that are exacerbated by both endogeneous and exogeneous emotional stimuli.

## Methods

**Fig. 1:**
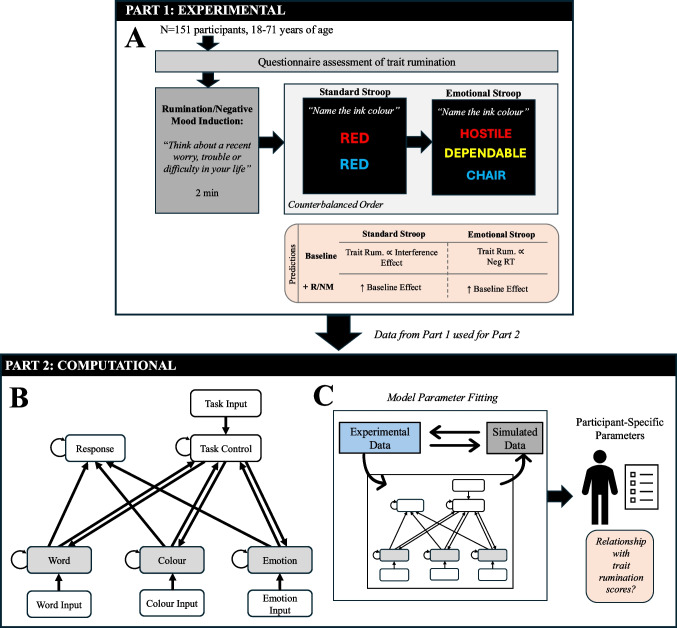
Outline of study methodology. **A** Experimental arm of study, including rumination questionnaire measures, a rumination/negative mood induction protocol, and the standard and emotional Stroop tasks presented in a counterbalanced order. We predict that the congruency effect will be proportional to an individual’s trait rumination levels, indicating inhibitory control deficits, that are exacerbated by the rumination/negative mood induction. We also predict that reaction times for negatively valenced words will be slower for individuals with high trait rumination, with the rumination/mood manipulation exacerbating this effect. **B** A computational model of the standard and emotional Stroop tasks (Cohen et al., 1990, p. 199). Bidirectional excitatory connections are indicated by arrowheads, and within-layer connections are all inhibitory. Grey boxes indicate hidden layers for word, color, and emotional semantic processing. **C** Model fitting procedure. Parameters of the computational model were numerically optimized, one participant at a time, to fit the model’s performance to the participant’s Stroop data from part one of our study. Model parameter values were then correlated with participant trait rumination scores

We used both experimental and computational approaches to investigate the impacts of bottom-up cues on inhibitory control in trait rumination (Fig. 1). Our experimental arm involved first measuring participant trait rumination, followed by assessing inhibitory control using the standard Stroop task and the effects of *exogenous* emotional cues using the emotional Stroop task. Both tasks were performed after a rumination/negative mood induction, a manipulation designed to increase *endogenous* bottom-up emotional cues. To study underlying computational mechanisms, we modified an existing parallel distributed processing-based model of the Stroop task and subsequently numerically fit the model parameters to individual participant Stroop data.

### Experimental design

To characterize the relationships between inhibitory control, trait rumination, and bottom-up cue processing, participants first completed a questionnaire measure of trait rumination, followed by a rumination/negative mood induction (Watkins, 2002), and the standard and emotional Stroop tasks (Riemann & McNally, 1995; Segal et al., 1995; Stroop, 1935, p. 19; Waters et al., 2005; Williams et al., 1996) presented in a counterbalanced order (Fig. 1A). The standard Stroop task is a common test of inhibitory control, as it requires the participant to override the predominant tendency to read words aloud when naming the ink color of words, particularly when the word name and ink color conflict (e.g., the word “green” printed in red ink). The emotional Stroop task presents words with positive, negative and neutral valence, enabling us to assess the impact of bottom-up exogenous emotional cues on performance as a function of trait rumination levels. The rumination induction manipulation was intended to enhance levels of endogenous bottom-up emotional cues (i.e., state rumination and/or negative mood), predicted to impact performance on both Stroop tasks, particularly when the latter is immediately preceded by the rumination/negative mood induction. All questionnaire measures and cognitive tasks were implemented through the PsyToolkit online platform (Stoet, 2010, 2017). We aimed to recruit a total of 140 participants (70 participants per counterbalancing condition), following sample size calculations from previous online Stroop experiments (Wielgopolan et al., 2025). Participants fluent in reading and writing in English were recruited world-wide from the Prolific crowdsourcing platform (www.prolific.com) with the following exclusion criteria: no previous history or current diagnosis of mental illness, head injury, or impaired color vision. We excluded individuals with a previous or current diagnosis of mental illness and head injury to mitigate psychological risks associated with the rumination induction procedure of our study. Participants provided informed consent prior to beginning the study, and all study procedures received ethics approval from the McMaster Research Ethics Board.

#### Questionnaire measures of rumination

We assessed trait rumination using the 22-item Ruminative Response Scale (RRS) (Nolen-Hoeksema & Morrow, 1991), which involved indicating what participants generally do with respect to rumination and depression-related statements (e.g. “I think about a recent situation, wishing it had gone better”) using a Likert scale from 1 to 4, where 1 is “almost never,” 2 is “sometimes,” 3 is “often,” 4 is “almost always.” As a post hoc decision, we centered our analyses around the brooding subscale of the RRS only, because this subscale may be better suited than the total RRS score to capture rumination as a maladaptive repetitive thought process relatively independent of depressive symptoms (Conway et al., 2000; Treynor et al., 2003). Additionally, the brooding, rather than reflection, score of the RRS has been predictive of depression severity (Treynor et al., 2003; Watkins, 2004).

#### Rumination/mood manipulation protocol

Prior to completing the two Stroop tasks, participants completed a rumination/negative mood induction protocol, where we asked them to think about a recent worry, trouble, or difficulty in their lives for 2 min, increasing endogenous emotional cues. This protocol was adapted from Watkins (2002) in which a similar method was used to induce a dysphoric mood. Along with negative mood, we believe that this approach may also be effective at inducing state rumination; indeed, previous research using momentary sampling has demonstrated that ruminative self-focus partially mediated the relationship between prior negative events (including “internal” negative events, i.e., thoughts) and momentary negative affect (Moberly & Watkins, 2008). Furthermore, cueing individuals to think of an unresolved goal also increases state rumination (Roberts et al., 2013). Taken together, we believe that our approach is valid for inducing rumination and/or a dysphoric mood, for the purpose of increasing endogenous emotional cues. In the context of the present study, previous researchers found that a different rumination induction increased the number of errors on the standard Stroop task in dysphoric individuals (Philippot & Brutoux, 2008). In light of this finding in depression, we hypothesised that a similar effect may occur in individuals with high rumination scores.

#### Stroop tasks

**Fig. 2:**
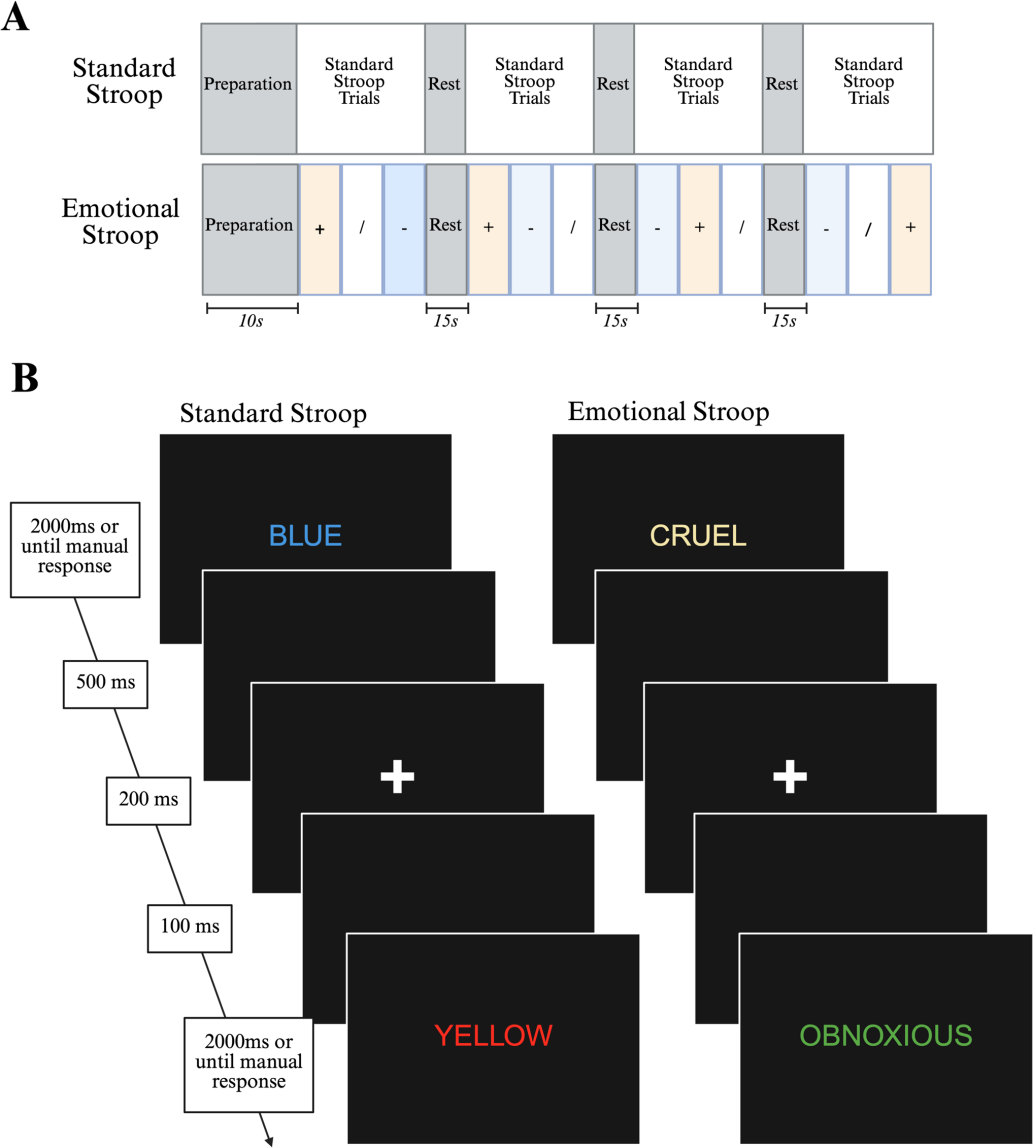
Block (**A**) and trial (**B**) timelines for standard and emotional Stroop tasks. Stimuli were presented in size 40 Arial font. Emotional Stroop trials were presented in the outlined subblocks of positively (+), neutral (/) or negatively (−) valenced words

Inhibitory control was assessed by using standard and emotional Stroop interference tasks (Riemann & McNally, 1995; Segal et al., 1995; Stroop, 1935, p. 19; Waters et al., 2005; Williams et al., 1996). For both tasks, participants identified the ink color of words and their reaction time was measured. There were four colors used (yellow, red, green, and blue), and participants indicated that the color of the word presented via manual response by using their keyboard (i.e., using the “y,” “r,” “g,” and “b” buttons, respectively). Participants were not explicitly instructed to use either their dominant hand only or both hands. All word stimuli appeared in all colors. The order in which the blocks of standard and emotional Stroop tasks were completed was counterbalanced between participants. Counterbalancing in this way allowed us to investigate standard and emotional Stroop performance immediately after the rumination/negative mood induction, providing us with insights on how endogenous emotional cues impact inhibitory control and exogenous emotional cue processing. The tasks performed second allowed us to measure inhibitory control and exogenous emotional cue processing after the rumination/mood manipulation was expected to subside. The first counterbalancing group (CB1) involved performing the standard Stroop first, followed by the emotional Stroop, and the second counterbalancing group (CB2) involved performing the emotional Stroop first, followed by the standard Stroop. Block and trial timelines are shown in Fig. 2.

In the standard Stroop task, ink color could be either congruent or incongruent with the word presented (e.g., the word “red” presented in red ink for congruent conditions or blue ink for incongruent). Participants completed four blocks of standard Stroop trials; each block contained 48 trials followed by a 15-s rest period. Participants were given a 2-s window within which to respond. We hypothesize that individuals with high brooding scores show greater Stroop congruency effects and larger increases in reaction time following the rumination/negative mood induction.

In the emotional Stroop task, a variant used in previous research (Segal et al., 1995), words varied in both their emotional valence and their ink colors. As with the standard Stroop task, participants had 2 s to indicate the ink color of the word presented. Participants completed four blocks of emotional Stroop trials; each block contained 16 positive, 16 negative, and 16 neutral words, followed by a 15-s rest period. These words were selected from their respective word banks in a random order without replacement. The words in our emotional Stroop task were drawn from a list of self-referential/personality trait terms with negative and positive (e.g., “hostile” and “sincere,” respectively) emotional valence (Segal et al., 1995) and neutral object words for the neutral valence condition (e.g., “chair”) (Battig & Montague, 1969). Specifically, 16 of the highest-rated positive and 16 of the lowest-rated negative words were chosen from a list of personality-trait words organized by likableness ratings (Anderson, 1968, p. 19), along with 16 neutral words chosen from a compilation of object categories (Battig & Montague, 1969). Words from the personality-trait word bank were chosen, because we hypothesised that these words may be particularly relevant for rumination, which is often self-referential (Watkins, 2004). We also assessed the emotional valence of these words via an objective measure by using the Valence Aware Dictionary and Sentiment Reasoner (VADER) tool (Hutto & Gilbert, 2014) from the Natural Language Toolkit (v. 3.8.1) (Bird et al., 2009) in Python (v. 3.9.13), a sentiment analysis tool that uses a set of predefined rules and a lexicon of words to determine emotional tone (Supplemental Table 1). This tool assigned a numeric valence score (i.e., a continuous value between − 1 and 1) to each word; a negative score indicated negative valence, and a positive score indicated positive valence. Scores close to zero indicated neutral valence. We used these scores in our subsequent analyses.

**Table 1:** Participant demographics. Counterbalancing group 1 completed the standard Stroop task first, immediately following the rumination induction, while counterbalancing group 2 completed the emotional Stroop task first

	Counterbalancing #1 (*n* = 76)		Counterbalancing #2 (*n* = 75)		Full sample (N = 151)	
	*n*	%	*n*	%	*n*	%
Sex						
Male	39	51%	42	56%	81	53.6%
Female	37	49%	33	44%	70	46.3%
Age						
18–35	56	74%	54	72%	110	72.8%
36–53	13	17%	18	24%	31	20.5%
54–72	7	9%	3	4%	10	6.6%

A systematic analysis of the Stroop task in depression reported slower reaction times for both positively and negatively valenced words (Epp et al., 2012), suggestive of a general (i.e., rather than valence-specific) attentional bias for exogeneous emotional stimuli. There may, however, be valence-specific *disengagement* deficits associated with rumination; indeed, trait rumination has been associated with slower disengagement from negative information (Grafton et al., 2016; Sanchez-Lopez et al., 2019). Therefore, similar to the standard Stroop experiment, we hypothesize that individuals with high brooding scores may demonstrate slower reaction times for negatively valenced words, with greater increases in reaction time following the rumination/mood manipulation.

### Computational model

The Stroop GRAIN (Graded, Random, Activation-based, Interactive and Nonlinear) model (Cohen & Huston, 1994; Cohen et al., 1990), which simulates the classic Stroop task, was extended to include an emotional semantic hidden layer and an emotional processing unit within the task control layer to facilitate the study of bottom-up influences on inhibition (Fig. 1B). To implement these extensions, we modified the base GRAIN model available through the *PsyNeuLink* simulation package (v. 0.15.1.0) (Cohen, 2016) in Python (v. 3.9.14). This model uses a connectionist (i.e., neural network) parallel distributed processing framework, where different stimulus qualities (word identity vs. ink color vs. emotional valence) are each processed by their own separate, parallel pathways. Automaticity of processing is dependent on the pathway strength, which increases with training. Attentional inhibition is facilitated by the strength of the top-down input from the task control layer to the hidden layers in this model. Top-down inputs from the task control layer represent cognitive “goals” (e.g., color naming, word reading, or emotional processing). A balance between automaticity and task demand characterizes attentional (i.e., inhibitory) control in this model. The original GRAIN model has been shown to simulate the time course of the potential psychological mechanisms underlying the Stroop congruency effect, along with the impacts of top-down, goal-driven behavior (Cohen et al., 1992).

Variants of this model have also simulated the enhanced Stroop congruency effect and emotional Stroop effects in depression (Siegle et al., 2004; Stolicyn et al., 2017). Manipulations to the task control layer, proposed to represent prefrontal control mechanisms (Cohen et al., 1992), were able to simulate the enhanced Stroop congruency effect in depression (Siegle et al., 2004). An alternative model assumed that depression may be associated with a conditioned response to negative stimuli and simulated the emotional Stroop task by incorporating layers representing the ventral tegmental area and amygdala. This model was able to simulate depression-related slow reaction times for negatively valenced stimuli (Stolicyn et al., 2017). Our simpler approach of adding a single layer for emotional semantic processing is appropriate for the present study, because we cannot apply the same assumptions regarding the neural circuitry underlying Stroop performance in depression to our study of inhibitory control in trait rumination. Although relatively more abstract, our model variant still incorporates enough detail to allow for the investigation of the mechanisms of bottom-up influences on inhibitory control.

#### Model architecture and components

A schematic of the model is shown in Fig. 1B. The architecture and theoretical thrust of this model has been described in depth by previous researchers (Cohen et al., 1990, 1992); we therefore present a brief overview. The input and hidden layers of the model consist of color, word, and emotion processing layers, comprising three neural “units” each (representing either the word or ink color for green, red, or black/blank stimuli; and negative, neutral, and positive for emotional valence), which receive inputs prior to task completion. A task control layer represents cognitive “goals” with units representing color naming, word reading, and emotional processing. There are bi-directional connections between the color, word, and emotional processing layers and the task control layer. Finally, a “response” layer encodes either a response of “green” or “red” and is connected to the color, word, and emotion processing layers with bidirectional symmetric weights. There are additionally within-layer inhibitory connections between each pair of units, facilitating lateral inhibition.

By virtue of this architecture, activation values of the units within the color, word, and emotion layers propagate to the higher-level layers, and the activation values within the task control layer propagate to the lower-level layers. Over time, a stable pattern of activity emerges, informing the activation levels within the response layer. Each unit’s activation at a given time,* t*, is defined as the running average of its inputs:

$${a}_{i,t }(t) = {a}_{i,t-1}(1-\tau ) + {net}_{i,t}\tau$$

where *a*_i,t_ is the activation of unit *i* at time *t*, and τ is the temporal integration rate. The integration rate determines how quickly synaptic inputs from the previous time point are integrated into the current activation value. Slow integration will therefore lead to the persistence of activity patterns over time. This is one of several free parameters in the model, along with its gain, bias, and weights (described below), that were numerically adjusted to fit the model performance to that of human participants. The net input at time *t* for unit *i*, *net*_*i,t*_, is described as follows:

$$ne{t}_{i,t} = {\sum }_{j}{a}_{j,t}(t){w}_{j,i}$$

where *a*_j,t_ is the output of unit *j* at time *t*, and *w*_j,i_ is the weight of the synapse from unit *j* to unit *i*. Each unit’s activation dynamics, and thus its outputs, are governed by a logistic function, ranging between 0 and 1, parameterized by its *gain* (*k*) and *bias* (*x*_*0*_):

$${a}_{i,t} =\frac{1}{1+{e}^{-k(ne{t}_{i,t}-{x}_{0})}}$$

A unit’s gain determines the steepness of the activation curve (i.e., impacting the neuron’s sensitivity to changes in input values), while the bias represents the “S”-shaped function’s inflection point. Changing the bias therefore “shifts” the curve either to the left (making the neuron more easily excitable) or right (making the neuron less excitable).

#### Simulated experiments

All Stroop simulated experiments began by initializing activations within the emotional semantic processing hidden and task control layers with binary input patterns corresponding to the upcoming task. To simulate the rumination/negative mood induction during this initialization phase, the following node activations were set to 1: the “negative” valence node within the emotion hidden layer, and the “emotional processing” and “color naming” nodes of the task control layer. To simulate the baseline condition, only the initial activation of the “color naming” node of the task control layer was set to an input value of 1. The remaining nodes’ initial activations in these layers were set to 0. The activations within the task control and emotion layers were clamped to these initial values while activity within the network settled across 50 cycles prior to beginning the experiments.

To simulate the standard Stroop task, incongruent and congruent input patterns were presented to the word and color processing hidden layers (i.e., the appropriate “red” and “green” processing node activations within each layer were set to input values of 1). To simulate the emotional Stroop task, the “negative,” “neutral,” or “positive” node activations within the emotional processing hidden layer corresponding to the input stimulus valence were set to 1. Unit activations were updated after each simulation cycle until 1 unit within the response layer crossed a threshold of 0.55. The number of cycles required to achieve a response (*RT*_*cycles*_) was then scaled using a regression parameter (*K*), representing the number of cycles that correspond to 1 ms, and an intercept (*I*), representing stimulus preprocessing/response execution time:

$$R{T}_{ms} = R{T}_{cycles}*K + I$$

*RT*_*ms*_ therefore represents the simulated reaction time in milliseconds.

#### Model fitting

Parameters within the model were numerically adjusted to fit the model’s performance to participant standard Stroop data collected in Part 1 of this study. Correlations between model parameter values and participant brooding scores may point toward the computational mechanisms underlying Stroop performance in rumination. Numerical fitting of these models allowed for an objective evaluation of the mechanisms underlying bottom-up influence on inhibitory control in rumination. The model fitting procedure may favor the task control → hidden layer pathway (i.e., top-down pathway facilitating inhibitory control), the hidden layer → task control pathway (i.e., the bottom-up pathway), or both; a bias toward one and/or the other of these two pathways would represent the relative influence of inhibitory control deficits and/or increased sensitivity to bottom-up cues in rumination.

The following parameters can be fit to participant Stroop data: top-down synaptic weights from the task control layer to the color/word and emotion layers; bottom-up synaptic weights from the emotion layer to the task control layer; integration rates within the emotion and task control layers; and unit biases and gains within the emotion and task control layers (Fig. 1). To evaluate whether fitting all parameters was necessary, we conducted a model comparison procedure to identify the model that best balanced model fit and complexity (quantified using the Bayesian Information Criterion) while minimizing parameter interdependence (quantified using pairwise Pearson correlations and mutual information between parameters). We outline these details further in the Supplementary Materials. We compared four models: 1) a *base* model in which only the GRAIN model weights were fitted; 2) a *weights* model, which added the task control ↔ emotion layer weights to those in the base model; 3) a *neuron* model, which added biases, gains, and integration rates within the task control and emotion hidden layers to the base model weights; and 4) a *full* model, which included all parameters described above.

We hypothesize that individuals with higher brooding scores, who we expect to show heightened congruency effects particularly following the rumination/negative mood induction, will exhibit model parameters reflecting stronger bottom-up synaptic weights relative to top-down pathways, as well as steeper unit gains in the emotion processing layer. This pattern is akin to the bottom-up hypotheses implemented in a previous Stroop model of conditioned response in depression (Stolicyn et al., 2017), capturing both increased sensitivity to emotional cues and greater emotional interference in top-down control. An evolutionary algorithm was used to fit these models, implemented using the *inspyred* (v. 1.0) (Tonda, 2020) package in Python. Further details regarding this procedure can be found in our supplementary materials. Briefly, evolutionary algorithms iteratively mutate, and evaluate the “fitness” of, different potential parameter sets (Kochenderfer & Wheeler, 2019). “Fitness” here was defined for each participant (*i*) as the following objective function:

$$Fitness_i=0.3\ast\left({Congruent}_{Data}-{Congruent}_{Sim}\right)^2+0.3\ast\left({Incongruent}_{Data}-{Incongruent}_{Sim}\right)^2+0.4\ast\left[\left({Incongruent}_{Data}-{Congruent}_{Data}\right)-\left({Incongruent}_{Sim}-{Congruent}_{Sim}\right)\right]^2$$

which is the weighted sum of three terms: the squared error between the simulated and mean experimental reaction times for 1) congruent trials and 2) incongruent trials, and the squared difference in congruency effects between model and data. The latter term characterizes the Stroop congruency effect, in which we would expect slower response times for incongruent trials relative to congruent trials. Without adding a term explicitly capturing this relative difference, we risked the algorithm converging to trivial solutions, where the model is fit well to either the congruent or incongruent condition, but not both. Adding this constraint reduced that risk, while ensuring that our fitted models captured individual differences in Stroop congruency effects. We also conducted a partial parameter recovery procedure to assess the stability and reliability of the selected parameter set, with details and results presented in the supplementary materials.

All simulations were conducted on a MacBook Pro M1 chip. Code for our simulations can be found in the following GitHub repository: https://github.com/selenasingh/StroopModel. Data from the experimental arm of our study is also available in the following repository: https://osf.io/7xbg8/overview.

### Analysis

#### Experimental Stroop data

The predicted impacts of brooding, trial condition (congruence and emotional valence), counterbalancing, age, sex assigned at birth, and the random effects attributable to individual differences on reaction time for the standard and emotional Stroop trials were characterized by using generalized linear mixed effects models (GLMM) assuming gamma distributed variables and an identity link function, following the recommendations made for analyzing reaction time data, which is inherently skewed (Lo & Andrews, 2015). Skewed data are typically transformed first before being analyzed with a linear model to ensure that the model’s normality assumptions are held. Transforming and scaling time-dependent variables, such as reaction time and age, may, however, obscure scale-dependent interactions (Lo & Andrews, 2015). Owing to this limitation, GLMMs have been recommended for the analyses of reaction time data (Lo & Andrews, 2015), because these types of statistical models relax the assumption that the dependent variable and residuals follow a normal distribution. Because we analyzed the standard and emotional Stroop data separately, we used a Bonferroni-corrected statistical significance threshold of ɑ = 0.025. The models are presented below in *R* syntax for the *lme4* package in the R programming language (Bates et al., 2015):

$$RT \sim counterbalancing*congruency*brooding + sex + age + \left(1\left|PID\right.\right)$$

$$RT \sim counterbalancing*valence\_score*brooding + sex + age + (1\left|PID\right.)$$

where *(1|PID),* in R syntax, indicates a random effects term for participant ID. For the emotional Stroop data, we used the valence scores for the negative, neutral and positive words.

As additional post hoc confirmatory analyses, we assessed group-level differences in reaction time across conditions by using nonparametric Friedman tests for standard and emotional Stroop trials. Where the Friedman test indicated significant differences, we performed pairwise Wilcoxon signed-rank tests with Bonferroni correction for multiple comparisons.

We also performed separate post hoc analyses for both standard and emotional Stroop tasks, accounting for individual differences in congruency effects in the standard Stroop task and potential language processing-related confounds in the emotional Stroop task. For the standard Stroop task, we refit the above mixed model using a random slopes structure for congruency (i.e., *(*1 + *congruency | PID)*, rather than *(1|PID)*) to capture within-participant variability in congruency effects for the standard Stroop data only. Indeed, individuals may vary in both their baseline reaction time and congruency effect magnitude. We report the results of these models alongside those from the original analyses. Given that the inclusion of a random slope increased model complexity, we also conducted simulation-based power analyses using the *simr* package in R (detailed in supplementary materials) to estimate the sample size required to reliably detect the three-way interaction under this model structure. For emotional Stroop post hoc analyses, we removed trials including the negative word stimuli “GREEDY” and “RUDE,” which share their first few letters with the colors “GREEN” and “RED,” introducing potential first, second, and third letter Stroop effects (Bibi et al., 2000). Additionally, we included English as a second language as a covariate in a GLMM statistical analysis of the emotional Stroop data to account for potential second-language processing differences (Fan et al., 2016; Lizarazo Pereira et al., 2023) (Supplementary Materials).

#### Parameters from computational model fitting

The predicted impacts of the normalized (i.e., z-scored) fitted parameters from the selected model on normalized participant brooding scores were characterized by using a linear model, using a statistical significance threshold of ɑ = 0.05. We describe this analysis in further detail after presenting the selected model in the *Results*, Sect. 3.2.

## Results

### Experimental results

#### Participant characteristics

A total of 155 participants were recruited worldwide from the Prolific crowdsourcing platform. Data from four participants were excluded due to missing questionnaire and/or experimental data. We analyzed standard Stroop data from 151 participants, and emotional Stroop data from 150 participants. The distributions of participants’ sex assigned at birth and age are shown in Table 1. Participant nationality data is presented in supplementary materials, Fig. 1; in summary, the majority of our participants were from South Africa (*n* = 30; 19.8%), the United Kingdom (*n* = 22; 14.6%), Poland (*n* = 20; 13.24%), Portugal (*n* = 18; 11.9%), Mexico (*n* = 10; 6.6%), Greece (*n* = 9; 5.9%), and Hungary (*n* = 9; 5.9%). Fewer participants were from Italy (*n* = 5, 3.3%), India (*n* = 4; 2.6%), and Canada (*n* = 3; 1.98%). There were two (1.32%) participants from each of the following countries: New Zealand, Nigeria, Puerto Rico, Spain, and Turkey. Additionally, there was one (0.66%) participant from each of the following countries: Bangladesh, Czech Republic, Estonia, France, Ireland, Israel, Latvia, Lesotho, Luxembourg, Romania, and Zimbabwe. While all of our participants were fluent in English, as per our inclusion criteria, the majority were nonnative English speakers (~ 60%, details in Supplementary Table 2).

#### Overview of standard and emotional Stroop performance

Descriptive statistics for standard and emotional Stroop trials are presented in Table 2. The standard Stroop task produced a congruency effect when averaging across all trials, with the majority of trials corresponding to correct participant responses (Mean RT_congruent_ = 805 ms, 95.2% correct; Mean RT_incongruent_ = 960 ms, 87.6% correct).

**Table 2:** Mean reaction time (RT) and accuracy data for standard and emotional Stroop trials

	Mean RT (ms)	SEM	95% BS-CI	% Correct	% Incorrect	% Overtime
Standard Stroop						
*Congruent* (n_trials_ = 14,443)	803	2.71	798–809	95.2%	2.46%	2.32%
*Incongruent* (n_trials_ = 14,473)	960	3.10	955–966	87.6%	9.20%	3.21%
Emotional Stroop						
*Negative* (n_trials_ = 9,664)	811	3.24	804–817	95.3%	3.03%	1.72%
*Neutral* (n_trials_ = 9,664)	822	3.36	815–828	94.3%-	3.69%	2.05%
*Positive* (n_trials_ = 9,589)	836	3.56	829–842	94.5%	2.96%	2.54%

*SEM* standard error of the mean; *BS-CI* bootstrapped confidence interval.

#### Rumination induction enhances Stroop congruency effect in relation to brooding scores

**Fig. 3:**
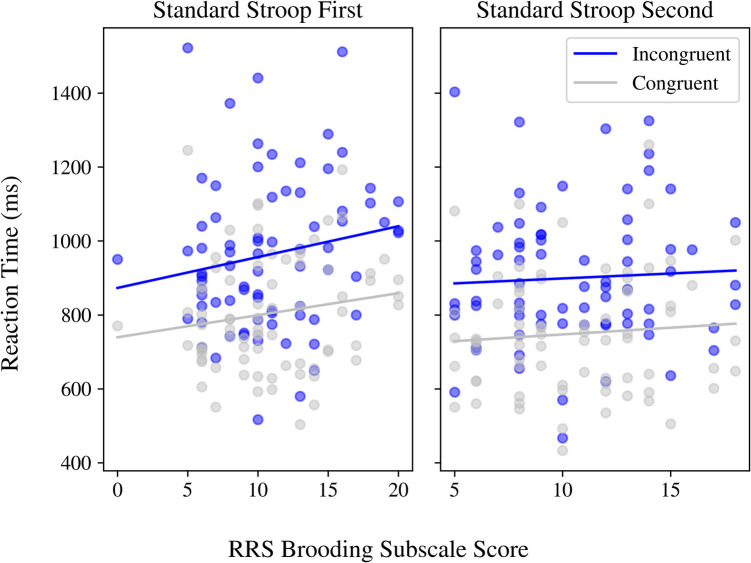
Standard Stroop task mean reaction times (ms) in relation to participant brooding scores (from the brooding subscale of the ruminative response scale [RRS]). Reaction times for incongruent trials increases when standard Stroop is completed immediately following rumination/negative mood induction (i.e., “Standard Stroop First”) for individuals with higher brooding scores

Regression statistics from our GLMMs for the standard and emotional Stroop tasks are presented in Supplementary Tables 3 and 4. Congruency significantly predicted reaction times, such that incongruent trials were associated with slower reaction times, regardless of counterbalancing condition or brooding scores (*congruency*: β = 89.69, confidence interval [CI] 30.45–148.92, *p* = 0.003) (Fig. 3). Brooding scores predicted the size of the Stroop congruency effect across participants (*congruency x brooding*: β = 7.47, CI 2.31–12.62,, *p* = 0.005), and this effect was moderated by task order (*counterbalancing* × *congruency* × *brooding*: β =  − 4.27, CI − 7.71 to − 0.82, *p* = 0.015) (Fig. 3). This interaction reflected a stronger association between brooding and Stroop congruency effects when the standard Stroop task followed the rumination induction (CB1; “Standard Stroop First”), a condition designed to increase endogenous bottom-up cues. In contrast, this association was attenuated when the standard Stroop task was performed after the emotional Stroop task (CB2). Together, these results suggest that endogenous bottom-up cues interfere with inhibitory control in trait rumination. As confirmatory analyses, the main effect of congruency was further supported by our Friedman test (ꭓ^2^(1) = 143.11, *p* = 2.2e-16). However, in our post hoc analysis using a GLMM including a random slope for congruency, none of these results remained statistically significant (Supplementary Table 5); this is likely due to our study being underpowered for this more complex model (Supplementary Fig. 2). Indeed, power analyses indicated that we would require ~ 1,100 participants to adequately power the three-way interaction between counterbalancing, congruency, and brooding, assuming a very small effect size of 0.05 (corresponding to ~ 20 ms), and α = 0.05. Nonetheless, the fixed effects estimates remained relatively unchanged between the random slope and original models (*congruency:* β_slope_ = 89.59, β_original_ = 89.69; *congruency x brooding:* β_slope_ = 7.48, β_original_ = 7.47; *counterbalancing x congruency x brooding:* β_slope_ =  − 4.28, β_original_ =  − 4.27).

#### Reaction times are not impacted by word emotional valence

**Fig. 4:**
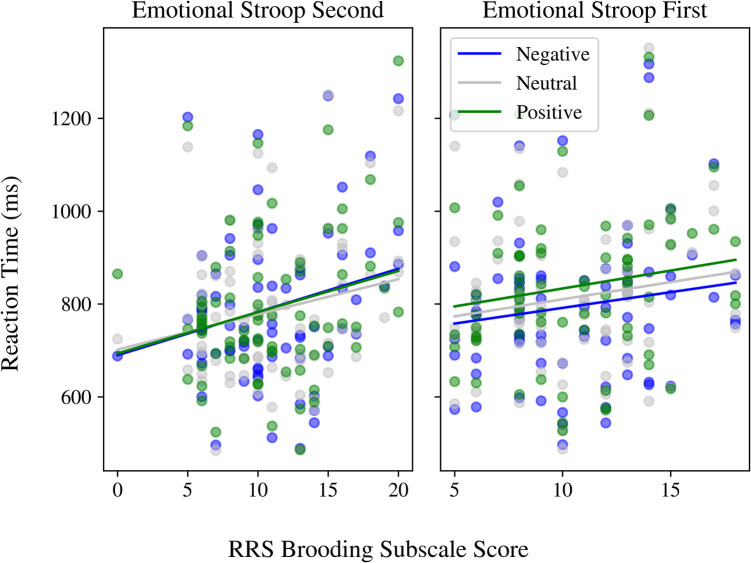
Emotional Stroop task mean reaction times (ms) in relation to participant brooding scores

Participant brooding scores, counterbalancing condition, and stimuli valence scores did not significantly predict reaction times for the emotional Stroop task using the GLMM (Fig. 4; Supplementary Table 4), suggesting that trait rumination may not be associated with valence-specific disengagement deficits, as originally hypothesized. Removing potentially confounding stimuli (i.e., “GREEDY” and “RUDE”) to mitigate Stroop first, second, and third letter effects did not impact these results (Supplementary Table 6). Adding English as a second language as a covariate post hoc significantly improved model fit (χ2(1) = 5.15, *p* = 0.023), revealing that nonnative English speakers were significantly faster on all trials compared with native English speakers (*ESL;* β =  − 67.77; CI − 127.21 to − 8.32; *p* = 0.025); however, the main effects of brooding, counterbalancing, and valence scores, and their interactions, remained insignificant (Supplementary Table 7). Interestingly, the Friedman test showed that reaction times differed significantly across valence conditions (χ2(2) = 17.05, *p* < 0.001). Pairwise Wilcoxon signed-rank tests revealed that reaction times differed significantly between positive and negative trials (*p* < 0.001) and between positive and neutral trials (*p* = 0.01). No significant difference was observed between neutral and negative trials (*p* = 0.085). These results suggest that, at the group-level, our participants had slower reaction times for positive trials over negative and neutral trials.

### Computational modelling results

We selected the *full* model for subsequent analyses as it demonstrated the best balance between goodness of fit, parsimony, and parameter independence relative to the other models (Supplementary Fig. 5). Although the *weights* model had the lowest summed (or “global”) BIC score out of the four compared models, it also produced parameter estimates that were highly correlated and interdependent (i.e., *r* = 0.71 between task control ↔ emotion hidden weights), limiting its utility for evaluating top-down and bottom-up mechanisms separately. The *full* model demonstrated a higher BIC score (i.e., the second lowest out of the four models), but substantially lower averaged absolute correlation and averaged pairwise mutual information between parameters (i.e., second lowest averaged correlation, and lowest mutual information) (Supplementary Fig. 5). Therefore, the *full* model was selected over the *weights* model for subsequent analyses. This model produced simulated results that align well with experimental Stroop data (congruent trials: R^2^ = 0.965, *p* < 0.001; incongruent trials: R^2^ = 0.976, *p* < 0.001) (Supplementary Fig. 3).

Our partial parameter recovery procedure demonstrated that only task control unit gains, biases and integration rates were fairly–moderately recoverable, with emotion semantic hidden layer parameters and model weights deemed unrecoverable (Supplementary Figs. 7 and 8). This points toward marked interdependence within hidden layer parameters and model weights, which is further supported by a hierarchical clustering analysis of parameters (Supplementary Fig. 6); our results should be interpreted as one possible solution explaining Stroop congruency effects with respect to ruminative brooding. We used this parameter set to identify the possible neural computations underlying impaired inhibitory control and sensitivity to endogenous bottom-up cues in trait rumination by assessing the relationships between participant brooding scores and fitted model weights, neuron-level parameters, and integration rates using a linear model. The model equation in *R* syntax for the *lm* function is shown below (R Core Team, 2023):

$$RRS\_brooding \sim emotion\_task\_w + task\_emotion\_w + task\_bias + task\_gain + emotion\_bias + emotion\_gain + cb*(task\_intg*emotion\_intg) + sex + age$$

where *emotion_task_w* and *task_emotion_w* represent the weights between the emotion hidden → task control layers, and task control → emotion hidden layers, respectively. Unit biases and weights along with integration rates (“*intg*”) for the task control layer and emotion hidden layer were also included. The impacts of the rumination/negative mood induction on integration rates, which quickly change in biological networks in response to changes in neurotransmitter levels (Barberis et al., 2011), was captured by the *cb* (“counterbalancing”) term.

#### Top-down and bottom-up weights

**Fig. 5:**
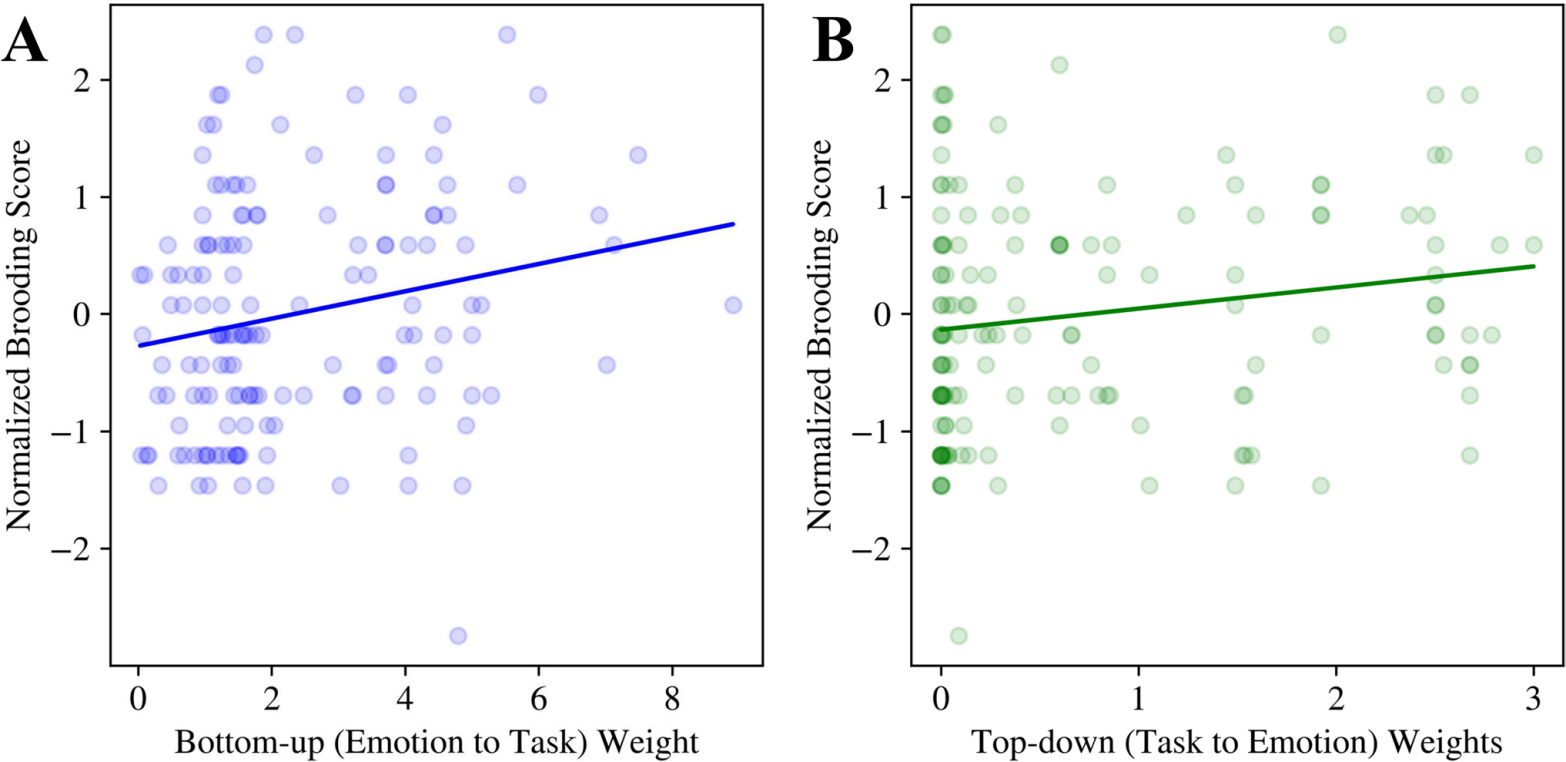
Normalized brooding scores in relation to fitted bottom-up (**A**) and top-down (**B**) computational model weights. Solid lines show the line of best fit. Emotion to task weights significantly predicted brooding scores, whereas task to emotion weights did not (see text for details)

Bottom-up, or emotion-to-task, fitted weights were positively and significantly correlated with normalized brooding scores (Fig. 5A) (*emotion_task_w*: *β* = 0.19, CI 0.04–0.34, *p* = 0.015). Higher brooding scores were therefore associated with larger weights between the emotion and task layer in our computational models, suggesting that individuals with higher brooding scores are more driven by bottom-up emotion cues. In contrast, top-down, or task-to-emotion, fitted weights were not significantly correlated with normalized brooding scores (Fig. 5B).

#### Task control and emotional semantic layer unit gains and biases

**Fig. 6:**
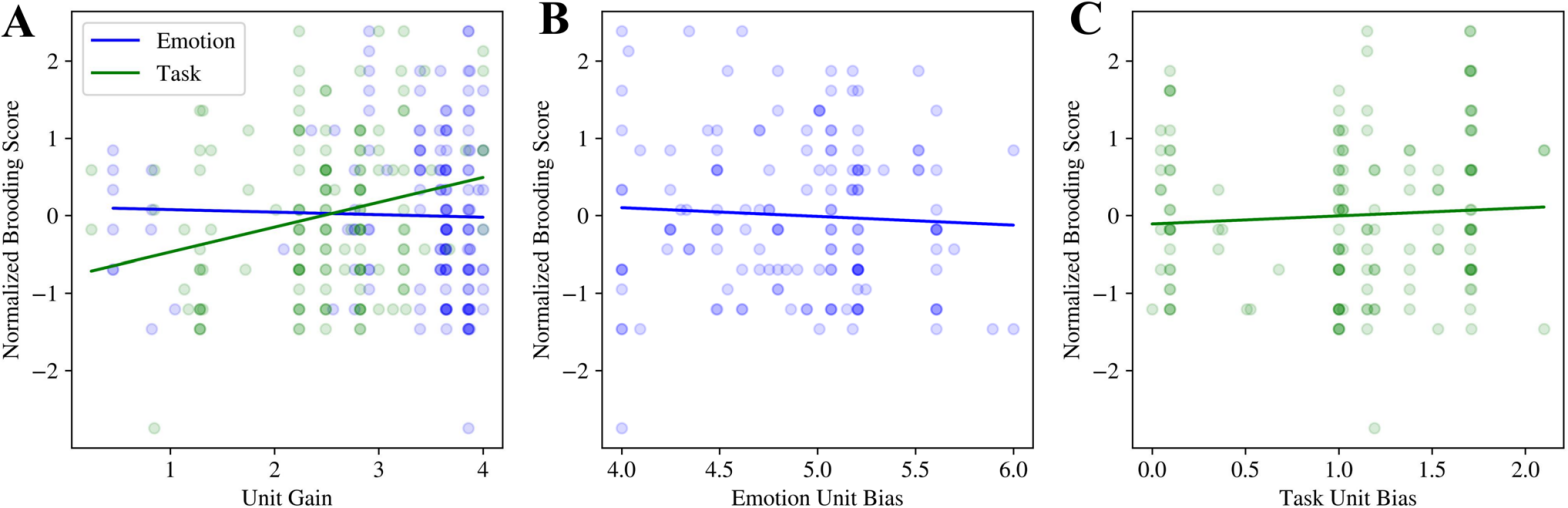
Unit-level parameters and their relationship with normalised brooding scores. **(A**) Activation function gain for emotion and task layer units. Task unit gain predicted normalised brooding score, whereas activation function bias for the units in the emotion layer (**B**) and task layer (**C**) do not predict normalised brooding scores

Results from our model fitting procedure for unit-level parameters are shown in Fig. 6. Unit gain values for the task layer predicted normalized brooding scores (*task_gain*: *β* = 0.26, CI 0.11–0.41, *p* = 0.001). Because a neuron’s gain determines how sensitive the neuron will be to changes in input, this result implies that individuals with higher brooding scores may be more sensitive to changes in task demands. In the context of the Stroop task, this would result in heightened interference effects due to greater instability in the task control layer, which is meant to maintain top-down goal-directed control over all hidden layers. Unit gain values for the emotional semantic processing layer, and unit biases for both task and emotion layers, did not have significant relationships with normalized brooding scores (Fig. 6).

#### Task control and emotional semantic layer integration rates

**Fig. 7:**
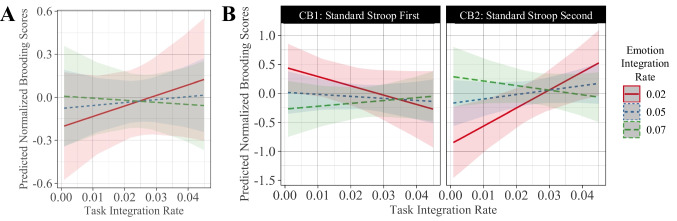
**A)** Task integration x emotion integration interaction: slower emotional integration and faster task integration predicted higher brooding scores, regardless of counterbalancing condition. **B)** Task integration x emotion integration x counterbalancing interaction: shifts in emotion and task layer integration rates after rumination induction predicted brooding scores. CB1: counterbalancing group 1, CB2: counterbalancing group 2. Slower emotion and task integration rates predicted higher normalized brooding scores only immediately after rumination induction (CB1), and not in the second counterbalancing condition (CB2)

Relationships between task control and emotional semantic hidden layer integration rates also predicted participant brooding scores (Fig. 7). Slower emotional integration with faster task control integration positively predicted participant brooding scores (Fig. 7A) (*task_intg* × *emotion_intg:* β = 0.64, CI 0.21–1.07, *p* = 0.004), meaning that trait rumination may be associated with slower temporal integration (i.e., increased persistence) of emotional cues yet faster temporal integration (i.e., faster activity decay) of task demand cues. Immediately after the rumination induction, slower integration in both emotional processing and task control layers predicted higher brooding scores (*counterbalancing* × *task_intg* × *emotion_intg*: β =  − 0.47, CI − 0.76 to − 0.19, *p* = 0.001) (Fig. 7B, panel CB1), reflecting that endogenous bottom-up cues reduced the speed of information integration within *both* task control and emotional processing layers, leading to the temporal persistence of both task control and emotional cues.

Given the significant three-way interactive effect of counterbalancing condition, task integration and emotion integration on brooding scores, we conducted a post hoc analysis evaluating, in each of the two counterbalancing conditions, the interaction between task control and emotion hidden integration rate. We used a Bonferroni-corrected ɑ = 0.025 to account for multiple comparisons. Detailed regression statistics can be found in Supplementary Tables 9 and 10. The interaction between task and emotion integration was not significant for the first counterbalancing condition (i.e., immediately after rumination induction). In contrast, in the second counterbalancing condition where the effects of the rumination induction are expected to subside, the interaction between task control and emotion hidden integration rates was significant (β =  − 0.33, CI − 0.56 to − 0.1, *p* = 0.005). Slower temporal integration in the hidden layer and faster integration within the task control layer may reflect a trait-level imbalance in activity persistence, regardless of endogenous bottom-up drive. These results reflect the differing impacts of bottom-up cues and trait rumination on the temporal nature of inhibitory control and emotional cue processing.

## Discussion

Our results indicate that inhibitory control is impacted in trait rumination, and is exacerbated by endogenous bottom-up cues, supporting our initial hypothesis for the standard Stroop task (Fig. 1). Our experimental Stroop results demonstrated that brooding scores were positively correlated with the Stroop congruency effect, i.e., participants with higher brooding scores were even slower on incongruent trials relative to those with lower brooding scores (Fig. 3). This effect was true regardless of counterbalancing condition, indicating baseline inhibitory control deficits in high trait rumination. Furthermore, this brooding and congruency relationship was heightened by the rumination/negative mood induction, reflecting the additional impacts of endogenous bottom-up cues on inhibitory control (Fig. 3). Our results agree with previous studies reporting inhibitory control deficits following a state rumination induction in depressed individuals (Philippot & Brutoux, 2008), while demonstrating that this effect is also relevant to trait rumination in healthy volunteers. Results from fitting a GLMM to the emotional Stroop task data demonstrate that there was no significant effect of the rumination/negative mood induction, stimulus emotional valence, or brooding on reaction time (Supplementary Table 4). However, pairwise Wilcoxon signed-rank tests support a trend towards slower reaction times for *positively* valenced stimuli across our entire sample. These results contradict our initial hypothesis that trait rumination may be associated with valence-specific disengagement deficits, reflected through slower reaction times for *negatively* valenced stimuli. This unexpected pattern may be a consequence of the word bank used, with the negative words simply not eliciting strong enough emotional arousal in our sample (we discuss this further below). Taken together, our results point towards inhibitory control deficits and an increased sensitivity to endogenous, rather than exogenous, emotional cues underlying trait rumination.

However, our experimental Stroop results warrant cautious interpretation, as our post hoc analysis, including a random slope for congruency, yielded substantially larger standard errors for our fixed effects, such that none of the key results remained statistically significant (Supplementary Table 5). Importantly, the fixed-effect estimates themselves did not change substantially. These results, alongside our power analyses, points toward 1) substantial within-participant variability in congruency effects, and 2) that our study is underpowered to detect the three-way interaction between counterbalancing, congruency, and brooding (see supplementary materials). Indeed our power analyses indicated that a larger sample size of ~ 1,100 participants would be required to achieve adequate power for such an analysis with a random slope for congruency, an unfeasible target given our present resources. We therefore encourage future work to account for variability introduced by within-participant congruency effects, particularly since an individual’s baseline reaction time may be related to congruency effects. Moreover, Stroop congruency effects may be susceptible to the reliability paradox, where high within-participant variability but low between-participant variability produces reliable group-level effects but limits test–retest reliability at the individual level (Hedge et al., 2018). As a result, even in a sufficiently powered study, estimates of random slopes for congruency effects may remain imprecise. Given that the original analyses were sufficiently powered for their aims (Supplementary Fig.  2 A), we proceeded with computational modelling to examine underlying mechanisms.

To identify the bottom-up and top-down neurocomputational mechanisms underlying the empirical standard Stroop effects, we numerically fit a parallel distributed processing-based neural network model of the Stroop task to these experimental data. This model fitting procedure allowed us to study the mechanisms of inhibitory control deficits at a deeper level than what may be afforded by behavioral experiments alone. The results from the *full* model implicate both bottom-up and top-down mechanisms underlying inhibitory control deficits associated with trait rumination. These mechanisms include steeper task control layer neuronal gains, increased bottom-up weights, and imbalanced temporal integration of emotional and task demand cues. Neuronal gain within the task control layer was positively associated with participant brooding scores across counterbalancing conditions (Fig. 6A). An increase in neuronal gain within the task control layer may reflect an increased sensitivity to changes in task demands, which in turn may lead to reduced cognitive flexibility, negatively impacting attentional disengagement and inhibitory control (the impacts of neuronal gain in the task control layer on the Stroop congruency effect are shown in Supplementary Fig. 4).

Brooding was also associated with faster temporal integration within the task control layer (Fig. 7); this may contribute to inhibitory control deficits by erroneously dampening the temporal window within which top-down task demands operate. Dampening activity persistence in this case may exacerbate interference effects; in contrast, having a persistent top-down influence on the rest of the network may *support* successful task completion for incongruent stimuli. Indeed, prefrontal activity persistence is critical for the maintenance of information in working memory (Riley & Constantinidis, 2016) and has been proposed to play a role in decision making processes (Curtis & Lee, 2010). Moreover, activity persistence in the anterior cingulate cortex has been implicated in sustained attention (Wu et al., 2017). Reductions in activity persistence, reflected by the faster temporal integration within the task control layer, may explain our results through the lens of poorer attentional or informational sustainment.

In addition to highlighting mechanisms for impaired inhibitory control, our computational modelling results also describe bottom-up influences, although these parameter estimates may be unstable (Supplementary Figs. 7 and 8). Our results demonstrate that the weights between the emotion hidden layer to the task control layer (Fig. 5), and slower temporal integration within the emotion hidden layer (Fig. 7), were each positively correlated with participant brooding scores. Notably, slower temporal integration of emotional cues intuitively reflects the persistent nature of rumination, and is consistent with the persistence of negative affect seen in those with high rumination (Blanke et al., 2022). From a neurobiological perspective, this result also aligns with the functional magnetic resonance literature demonstrating increased and sustained amygdalar activation in rumination (Knight et al., 2019; Mandell et al., 2014; Siegle et al., 2002). Additionally, the strengthened bottom-up connections from emotional inputs may explain the consistently observed increased default mode network connectivity in rumination (Zhou et al., 2020), with some studies indicating increased connectivity between the amygdala and inferior gyrus and precuneus (Feurer et al., 2021). Alternatively, it has been proposed that increases in neuronal gain enhance learning and decision making for dominant processing pathways, in that a steeper neuronal activation function leads to more efficient processing along pathways with stronger connections (Eldar et al., 2013). From this perspective, steeper neuronal activation functions within the task control layer (Fig. 6A) combined with increased bottom-up weights (Fig. 5A) may lead to the amplified influence of emotional cues on cognitive control. Although promising, these results pertaining to bottom-up mechanisms require further validation, as discussed later in the strengths and limitations section.

### Our results in the context of existing neurocognitive and computational theories of rumination

Along with describing bottom-up influences on inhibitory control in rumination, our results also support facets of the resource allocation hypotheses of rumination. Our post hoc analysis of the interactive effects of emotion and task control temporal integration rates on brooding in each of the two counterbalancing conditions revealed that this interaction was significant in the second counterbalancing condition but not immediately after the rumination induction. State rumination may therefore lead to a shift in cognitive or neurobiological resources responsible for this statistical effect, however, further research is warranted to understand the relationship between state rumination and the temporal persistence of neural emotional and attentional activity. From a neurobiological perspective, these changing resources may include neuromodulators that influence synaptic receptor dynamics, such as the slow dynamics of NMDA receptors involved in working memory related activity persistence within the prefrontal cortex (Van Vugt et al., 2020; Wang, 1999; Wang et al., 2013).

It is important to emphasize that while we have not explicitly modelled *rumination* per se, we developed individualized models (in those with a range of brooding scores) of the top-down and bottom-up processes involved in attentional inhibition; we were then able to assess what aspects of these models best explained participant brooding scores. In contrast, rather than fitting models to participant behavioral data, prior models have explicitly simulated the potential mechanisms of rumination (e.g., as a process arising from the continuous and interdependent evolution of emotion and cognitive processes) in a purely theory-driven fashion. We now review how our results align with these prior models. The first reported mechanistic model of rumination incorporated a constrained form of mind wandering (Van Vugt et al., 2018). The authors hypothesized that rumination arises from maladaptive habits of thought and modeled this as associative structures between memory “chunks.” The spread of activation from emotion to memory units within these chunks was thought to drive ruminative memory recall in this model, which is broadly compatible with our findings of increased emotional temporal integration (Fig. 7) and increased bottom-up weights in those with high brooding scores (Fig. 5). This model captured aspects of biased memory retrieval for negatively valenced memories, which aligned qualitatively with results from individuals with depression (Kaiser et al., 2018), and other models of attentional biases in rumination and depression (Siegle, 1999). A different model employed a dynamical systems framework to study the abstract moment-to-moment interactions between and temporal behavior of contextual demand, attentional selection, working memory, and affect, simulating state rumination as a passive process that arises during conditions of low contextual demand and intrinsic negative affective bias (Amir & Bernstein, 2022). In contrast, we demonstrated that trait rumination was associated with broader cognitive control deficits even in situations with high contextual demand (i.e., performing a Stroop task), suggesting that the mechanisms of rumination impact cognition independent of contextual demand. Framing rumination as a form of passive mind wandering during states of low contextual demand may therefore fail to capture rumination mechanisms that lead to broader cognitive deficits.

Both of the dynamical models of rumination discussed above include a negativity bias as one mechanistic component of rumination. As previously mentioned, results from our emotional Stroop task did not show a statistically significant interactive effect of word valence and brooding scores on reaction time (Fig. 4; Supplementary Table 4). This null result may be attributable to a number of factors. First, we conducted this experiment in healthy volunteers without a previous mental health disorder and assessed rumination using the brooding subscale of the RRS rather than the composite score. The negativity bias previously reported in rumination may, in fact, be primarily associated with *depressive* symptomatology (Dalgleish & Watts, 1990), which the composite RRS score has been criticized to also measure (Treynor et al., 2003). Second, the stimuli used for our emotional Stroop experiment may not evoke sufficient emotional arousal in our participants. This may be explained by second-language processing effects, as evidenced by our post hoc analyses including English as a second language as a covariate (Supplementary Table 7); indeed, nonnative English speakers were faster on all trials than their native English speaking counterparts. Because second-language processing may be more effortful (Grey, 2023; Zheng & Lemhöfer, 2019), nonnative English speakers may find it easier to focus on stimuli color by avoiding the additional cognitive load of engaging in semantic processing. Native English speakers, on the other hand, may process the semantic content of the stimuli in a more “automatic” fashion (Favreau & Segalowitz, 1983), leading to valence-related slow-down effects. To address this in greater detail, future work should consider second-language (L2) proficiency and age of acquisition as additional covariates. Moreover, as we have an international sample, the cultural and societal context will indeed influence the relationship between emotional valence and arousal (Kuppens et al., 2017; Lim, 2016; Yik et al., 2023). The word bank we used was originally designed for a study in depressed Canadian patients and healthy controls (Segal et al., 1995). Future studies of the emotional Stroop task in an international sample should consider asking participants to provide a list of personally meaningful words, accompanied by their self-reported negative vs. positive valence and arousal scores.

### Strengths, limitations and future directions

#### Experimental design

Our experimental design has a number of strengths while also introducing potential limitations. Strengths include the use of an international sample and analyzing continuous emotional Stroop valence scores. Many psychological experiments draw on participant samples with narrow demographic characteristics, such as undergraduate students. By recruiting individuals world-wide from a wide range of ages, and having a balanced sample between sexes, our results may be more generalizable than studies with samples that are restricted geographically. Objectively assessing the emotional valence of the words within the word bank for our emotional Stroop task using a sentiment analysis tool is another strength of our study. Prior studies tended to use subjective three-valued categorical ratings of emotional valence (Segal et al., 1995). Our sentiment analysis procedure assigned each word a numerical score ranging continuously from − 1 to 1, representing continuous negative and positive valences respectively (Supplementary Table 1). Using these raw valence scores in our analysis of the emotional Stroop task allowed for a more fine-grained numerical analysis of the impacts of emotional valence on reaction time (Supplementary Table 4). Conversely, subjective valence ratings have the advantage of being individualized. As noted above, this can be advantageous in a diverse international sample such as ours.

A limitation of our study design is that our measure of rumination, the RRS (Nolen-Hoeksema & Morrow, 1991) may be confounded by depression symptomatology (Treynor et al., 2003). Although we mitigated this by using the brooding subscale only, adding another scale that measures perseverative thinking tendencies exclusively (Ehring et al., 2011), and including a validated measure of depressive symptomatology as a covariate in our statistical analyses, may be worthwhile. The addition of such scales would be especially important when extending this study to a clinical population, as the brooding and reflection RRS subscales may not be dissociable in depressed patients (Whitmer & Gotlib, 2011).

Additionally, our rumination induction may not be relevant for all forms of repetitive negative thinking and may be better suited for inducing anxious worrying rather than the more abstract repetitive self-referential thinking commonly seen in depression. We chose this procedure for increasing endogenous bottom-up cues by inducing dysphoric mood (Watkins, 2002), which we expect would increase state rumination (Moberly & Watkins, 2008; Roberts et al., 2013), following previous studies of the emotional Stroop task in depression (Philippot & Brutoux, 2008), although future work should indeed consider 1) validating our procedure using a within-experiment measure of state rumination (e.g., the brief state rumination inventory (Marchetti et al., 2018)), and 2) using other rumination induction procedures. Indeed, some have directly asked participants to first list and then dwell on their ruminative thoughts and/or images (McLaughlin et al., 2007), whereas others have asked participants to reflect on the consequences of their performance on cognitive tasks and social behaviors (Shull et al., 2016)*.* These alternative procedures may induce a broader range of repetitive negative thinking independent of negative mood than the procedure we chose. Future work should also consider including a sham condition as a control comparison to determine whether the increases in reaction time are specifically attributable to an increase in endogenous emotional cues or due to distraction.

#### Modelling approach

One major strength of our study design is combining experimental data with a mechanistically informed computational model. In previous work, we developed criteria to assess the face, predictive and construct validity of computational models (Nunes et al., 2022) following the validity criteria widely used for animal models (Belzung & Lemoine, 2011). Face validity concerns a model’s ability to simulate a clearly operationalized real-world feature of interest, which in our case is the Stroop congruency effect in relation to rumination. We supported our model’s face validity by directly fitting our models numerically to experimental data, by conducting a model comparison procedure to select a model that best balances complexity, fit and parameter independence, and by including a validated measure of rumination. We believe that we have presented the first study to numerically fit the Stroop GRAIN model to experimental data, allowing us to objectively evaluate cognitive control in rumination; previous models using variants of the GRAIN model have manually tuned parameters to align model outputs with data from individuals with depression (Stolicyn et al., 2017), risking experimenter bias. Additionally, construct validity concerns whether a model’s architecture is homologous to biologically or psychologically plausible mechanisms governing the system’s behavior. Our model also demonstrates construct validity as it embodies the cognitive theory of competitive pathways governing automatic and controlled processes (Cohen et al., 1990, 1992), allowing us to study bottom-up and top-down influences on inhibitory control. In contrast, other simpler models that also simulate decision making, such as drift diffusion models, may not be as informative as they are missing mechanistically relevant architectural components. Predictive validity concerns whether relevant transitions (e.g., the transition in and out of rumination, or the effects of a treatment) have been modelled and whether those transitions then predict corresponding transitions in the real-world; this remains to be investigated in future work. Interesting, repetitive transcranial magnetic stimulation (rTMS) to the left dorsolateral prefrontal cortex effectively reduces rumination (Chu et al., 2023) and improves inhibitory control (Corlier et al., 2020) in depression. Future work can use computational modelling in this context to predict whether the improvements in inhibitory control, via rTMS-mediated changes in functional connectivity within prefrontal-limbic circuits, underlie reductions in rumination levels.

We focused exclusively on mechanisms of inhibitory control in rumination in the present study, however we do recognize that attentional scope may also play a complementary role in rumination. The model-fitting approach we took here can be applied to study mechanisms of the attentional scope model of rumination (Whitmer & Gotlib, 2013), by using an existing model of attentional selectivity, such as the Shrinking Spotlight model, previously used in depression research (Grange & Rydon-Grange, 2022). Although this model primarily captures attentional selectivity, it does include parameters that capture the initial width of the attentional distribution, and the rate by which this distribution narrows around a stimulus of interest; both of these parameters have promise for computationally evaluating attentional scope in rumination if numerically fit to data from an attentional selectivity task (such as flanker task data: e.g., (Pe et al., 2013)).

Our model and experimental design should also be extended to address several additional features relevant to the two Stroop tasks. First, future work should consider the interdependence between the “word reading” and “emotional processing” pathways. Indeed, emotional salience may only be extracted after the word has been at least partially processed. Introducing a gating mechanism within the emotional semantic hidden layer that only allows for processing once the word processing layer crosses a threshold is a possible mechanistic implementation to address this. This model extension could provide insights on why nonnative English speakers were faster on emotional Stroop trials, as our data revealed here (Supplementary Table 7). Secondly, our Stroop model differs from the proactive-control task-conflict (PC-TC) model, which includes a “reactive” control mechanism (Kalanthroff et al., 2018), resolving poststimulus task conflict. Proactive control refers to priming word reading or color naming pathways, as we have implemented in our model, while the reactive control (or task-conflict) mechanism engages in cases of weak proactive control. The PCTC model has explained the Stroop reverse-facilitation effect, where participants may respond faster for neutral rather than congruent stimuli. Indeed, neuronal gain within the task control layer may impact reactive control (Tromp et al., 2022), which may be particularly relevant for rumination given the results we present here (Fig. 5A). We therefore encourage future work to implement an inhibitory pathway from the task control layer to the response layer, mirroring the PC-TC model (Kalanthroff et al., 2018) to assess proactive vs. reactive control in rumination. Finally, future work should consider alternating between emotional and standard Stroop trials, to assess the temporal dynamics that underlie emotional processing and inhibitory control through “spillover” effects from emotional Stroop stimuli on inhibitory control, as has been done by previous researchers (Kalanthroff et al., 2016; Straub et al., 2022, 2024).

Although promising, there are inherent limitations to any model fitting procedure. Model fitting procedures may produce degenerate solutions, in which multiple parameter sets can produce the same simulated behavior. Degeneracy is common in biologically inspired computational models (Huang et al., 2025; Marder & Taylor, 2011), and indeed in the brain, where different channel types and/or synaptic pathways give rise to similar electrophysiological behavior (De Vico Fallani et al., 2012; Marder & Goaillard, 2006; Mishra & Narayanan, 2021). Our partial parameter recovery procedure revealed that emotion hidden layer parameters and model weights were not recoverable (Supplementary Figs. 7 and 8), indicating such degeneracy. This is likely due to high interdependence among model weights and nonlinear relationships between the neuron-level parameters (Supplementary Fig. 6). Accordingly, the results presented here should be regarded as *one possible* solution explaining Stroop congruency effects in rumination, rather than the definitive parameter set. In future work, these interdependent parameters should be linked or reparameterized to improve identifiability. Given the nonlinear dependencies among neuron-level parameters, a detailed parameter sensitivity and dependency analysis will be required to inform the reparameterization strategy. Moreover, models should also be fit repeatedly using different hyperparameters, with the most probable solution selected from the resulting set, while also leveraging trial-by-trial variability to constrain model fits. Such an approach was infeasible given our present computational resources. Future work can first reparameterize the model to reduce interdependencies, then evaluate which hyperparameters and initial conditions yield the best-fitting solution using a model selection procedure, and finally conduct a thorough parameter recovery procedure to assess the reliability and stability of that solution.

Biological plausibility can always be called into question with any model. While the parallel distributed processing architecture of our model is inspired by the cognitive computational neurosciences, it remains relatively abstract. To increase biological plausibility, anatomical and physiological details could be incorporated (e.g., how heterogeneity in cell types impacts neural computations of inhibitory control). Our modelling results do align, broadly speaking, with previous evidence demonstrating persistent prefrontal cortex activity involvement during Stroop task performance (Banich et al., 2000a, 2000b; Parris et al., 2019; Zysset et al., 2001), which may be represented by our task control layer. The standard Stroop task has, however, also been associated with increased activation in the dorsolateral prefrontal cortex for task-irrelevant information during incongruent trials, suggesting that attentional selection may modulate the processing of task-irrelevant information in the Stroop task (Banich et al., 2000a, 2000b). Our model can indeed be extended to account for these data, following previous researchers (Herd et al., 2006).

From a biophysical perspective, several neuromodulators (e.g., acetylcholine, dopamine, noradrenaline and serotonin) are known to modulate attentional control (Thiele & Bellgrove, 2018) and may also be involved in cognitive flexibility (Aznar & Hervig, 2016). Because the pharmacological treatment of depression and anxiety commonly involves these neuromodulators, it would be valuable to incorporate their roles into a biophysically realistic (i.e., “spiking”) model of attention and rumination. Such a spiking model could additionally account for electroencephalography data which our relatively abstract model cannot. Rumination and inhibitory control have indeed been linked to interactions between high- and low-frequency neural oscillatory activity (Chen et al., 2024; Hwang et al., 2014; Wang et al., 2022). Investigating how top-down and bottom-up influences in rumination impact the computations performed by these neural oscillations within distributed networks is another open question.

### Conclusions

We present the first study to numerically fit a classical model of the Stroop task to experimental Stroop data to objectively evaluate bottom-up factors contributing to inhibitory control deficits in trait rumination. Our experimental results suggest that rumination is indeed associated with slower reaction times for incongruent stimuli, which is exacerbated by endogenous bottom-up cues, suggesting that trait rumination may be associated with both inhibitory control deficits and a sensitivity to endogenous emotional stimuli. The parameters derived from our model fitting procedure subsequently suggested that those rumination-related deficits may be attributable to steeper activation functions in task-control regions (leading to an increased sensitivity to changes in task demands) and stronger bottom-up weights. Our model parameters also suggest that there may be an imbalance in activity persistence within task control and emotion processing circuits in those with high trait rumination. Extending this work to a clinical population could provide insights on how the computational processes underlying inhibitory control in the context of rumination differ from processes in healthy volunteers.

## Supplementary Information

Below is the link to the electronic supplementary material.

Supplementary file1 (DOCX 8282 KB)

## Data Availability

De-identified data from this study are available through the following Open Science Framework repository: https://osf.io/7xbg8/overview

## Code availability

Code used for our simulations can be found in the following GitHub repository: https://github.com/selenasingh/StroopModel
