# Supplementary material for: Emotional modulation of inhibitory control in rumination from empirical and computational perspectives
Source: Cogn Affect Behav Neurosci. 2025 Oct 31;26(1):64–88. doi: 10.3758/s13415-025-01360-7 (PMC12847145; doi:10.3758/s13415-025-01360-7)
Supplement: Supplementary file 1 — Supplementary file1 (DOCX 8282 KB) [file 13415_2025_1360_MOESM1_ESM.docx]

***Supplementary Materials for:***

**Emotional modulation of inhibitory control in rumination from empirical and computational perspectives**

**Short Title:** Emotion, Rumination, and Stroop Interference

Selena Singh^a^, Benjamin Li^a^, Serenna Gerhard^b^, Abraham Nunes^c,d^, Suzanna Becker^a,*^

a) Department of Psychology, Neuroscience and Behaviour; McMaster University, Hamilton, Ontario

b) Sunnybrook Research Institute; North York, Ontario

c) Department of Psychiatry, Dalhousie University, Halifax, Nova Scotia, Canada

d) Faculty of Computer Science, Dalhousie University, Halifax, Nova Scotia, Canada

# 1. Supplementary Methods

## 1.1. Numerical Optimization-Based Fitting of Computational Models to Stroop data

To develop Stroop GRAIN models for each participant, a custom evolutionary algorithm was built using the *inspyred* (v. 1.0) and *PsyNeuLink* (v. 0.15.1.0) Python packages to facilitate parameter optimization, such that the resulting models simulated participant Stroop data. Evolutionary algorithms perform parameter optimization by iteratively mutating, then evaluating the “fitness” of a parameter set. Alternative parameter fitting procedures may update parameters with the goal of minimizing a gradient function. Gradient-based methods may lead to local minima solutions, and may not be ideal for finding the optimal solution for complex high-dimensional systems such as our computational model. Thus, an evolutionary algorithm is better suited for model fitting in our case.

Based on an initial parameter exploration, we set minimum and maximum parameter values that avoided asymptotic solutions (i.e., some increases in parameter values lead to exponential increases in reaction times). We deemed each model fit to be satisfactory if the simulated reaction times fell within 5% of the empirical reaction time, and 180 evaluations were made to ensure stable convergence to a minimum mean squared error value. Code for our model fitting procedure can be found in the following repository: https://github.com/selenasingh/StroopModel.

### 1.1.1. Model comparison procedure

To identify the most appropriate model architecture for subsequent analyses, we compared four variants of our adapted GRAIN model that differed in the inclusion of top-down and bottom-up mechanisms during model fitting: 1) fitting only the original GRAIN model weights (i.e., between task control, word, colour and response layers), 2) adding top-down (task control→emotion hidden) and bottom-up (emotion hidden→task control) weights to the base model, 3) the base model with neuron-level parameters only (gains, biases and integration rates within the task control and emotion hidden layers) and 4) a full model that includes fitting all weights and neuron-level parameters. Models were named as follows: *h0* for the base model, *weights* for the weights-only model, *neurons* for the model including neuron-level parameters, and *full* for the model that includes both weights and neuron-level parameters.

Model comparison was based on two criteria: 1) the Bayesian Information Criterion (BIC) to assess fit while penalizing complexity, and 2) the averaged absolute pairwise correlation and the averaged normalized mutual information between estimated parameters across participants, as measures of parameter dependencies. While BIC identifies the most parsimonious model in terms of fit and complexity, we also prioritized parameter interpretability. Specifically, low interdependence among parameters is critical for disentangling the contributions of top-down and bottom-up mechanisms. High correlations and/or mutual information between these parameters reduce identifiability and obscure mechanistic interpretation. Thus, the final model was selected based on the best balance between model fit (low BIC) and parameter interdependence (low average parameter correlation and mutual information).

A global BIC score was computed for each model by summating individual BIC values across participants. While the full BIC includes a curvature penalty based on the parameter-space Hessian, this is impractical to estimate for our complex, irregular models fitted only once. We therefore used the Gaussian special case that assumes independent and normally distributed model errors (Cavanaugh & Neath, 2019; Priestley, 1982):

$$BIC = n *ln(\frac{SSE}{n}) + k*ln(n)$$

Where *SSE* is the sum of squared errors between model output and participants’ Stroop data, *n* is the number of observations, and *k* is the number of free parameters. Mutual information is a measure of how much information one can obtain from one variable while observing another, and is thus a measure of mutual dependence sensitive to *both* linear and nonlinear relationships. We included mutual information as a criterion since neuron-level parameters may interact nonlinearly; a relationship that may be missed by Pearson correlations. To compute normalized mutual information, we binned parameter values into 10 equal-frequency categories (i.e., deciles), then computed the difference between their joint entropy and the sum of their individual entropies. The result was then normalized such that 0 represents complete independence while 1 indicates complete dependence.

### 1.1.2. Parameter Recovery

Our primary goal in the model fitting procedure was to evaluate competing mechanisms objectively, and at a finer resolution than what is afforded by behavioural data alone, for hypothesis-generating purposes. Given that we fitted multiple parameters to only three data points (mean reaction times for incongruent and congruent trials, plus the magnitude of the congruency effect), the solution space is inherently degenerate: different parameter sets can produce the same behavioural output. Despite this, we selected a model that balanced goodness of fit with parsimony while minimizing parameter interdependence. Best practice in such contexts is to conduct a parameter recovery procedure to assess the reliability of parameter estimates. In these procedures, the model is fitted to data generated from known parameter values, and recovery is quantified by comparing the recovered parameters to the known ones. We conducted a partial parameter recovery procedure, adapting it in two ways to account for both computational resource constraints and model-related limitations.

Firstly, we used a covariance matrix adaptation evolution strategy (CMA-ES) rather than the original evolutionary algorithm. CMA-ES adapts the search distribution’s covariance matrix based on the geometry of the solution space after each iteration, which makes it efficient at finding optimal solutions for complex optimization problems. We used the same minimum and maximum parameter bounds as the original algorithm. Our original evolutionary algorithm does not include covariance matrix adaptation, which meant it allowed for broad, unbiased exploration of the solution space with the tradeoff of being computationally expensive as it required a much higher number of evaluations (~250 for the original algorithm, whereas CMA-ES required only ~50-80 iterations to converge).

Secondly, given the degeneracy of the solution space, we fixed subsets of parameters to their originally-fitted values and attempted to recover the remaining ones using CMA-ES. Recovery on all parameters at once led to poor recovery (i.e., r < 0.5) for each parameter. Therefore, we first fixed all model weights and fit neuron-level parameters (integration rates, biases and gains), and then fixed neuron-level parameters and fit model weights (task→emotion and emotion→task). Due to resource limitations, we re-fit parameters only once from a single set of initial conditions rather than multiple times as in standard recovery procedures. We classified the quality of recovery using the correlation coefficients between original and recovered parameters, with poor recovery defined as r < 0.5, fair recovery as 0.5 < r < 0.75, good recovery as 0.75 < r < 0.9 and excellent for r values > 0.9, following previous work (White et al., 2017).

We also outline avenues for future work aimed at reducing solution degeneracy, thereby improving the feasibility of full parameter recovery. We present correlation matrices and dendrograms based on both Pearson correlation and normalized mutual information, illustrating linear and nonlinear dependencies among parameters in our selected model in Supplementary Materials section 2.5 below.

*1.2. Word bank and valence scores for emotional Stroop task*

*Table 1: Word bank and valence scores for emotional Stroop task.*

| **Word** | **Category** | **Composite Semantic Analysis Score** |
| --- | --- | --- |
|  |  |  |
| SINCERE | POSITIVE | 0.4019 |
| HONEST | POSITIVE | 0.5106 |
| UNDERSTANDING | POSITIVE | 0 |
| LOYAL | POSITIVE | 0.4767 |
| TRUTHFUL | POSITIVE | 0.4588 |
| TRUSTWORTHY | POSITIVE | 0.5574 |
| INTELLIGENT | POSITIVE | 0.4588 |
| DEPENDABLE | POSITIVE | 0 |
| THOUGHTFUL | POSITIVE | 0.3818 |
| CONSIDERATE | POSITIVE | 0.4404 |
| RELIABLE | POSITIVE | 0 |
| WARM | POSITIVE | 0.2263 |
| KIND | POSITIVE | 0.5267 |
| FRIENDLY | POSITIVE | 0.4939 |
| UNSELFISH | POSITIVE | 0 |
| HUMOROUS | POSITIVE | 0.3818 |
| LIAR | NEGATIVE | -0.5106 |
| PHONY | NEGATIVE | 0 |
| MEAN | NEGATIVE | 0 |
| CRUEL | NEGATIVE | -0.5859 |
| DISHONEST | NEGATIVE | -0.5719 |
| UNTRUTHFUL | NEGATIVE | 0 |
| OBNOXIOUS | NEGATIVE | -0.4588 |
| MALICIOUS | NEGATIVE | 0 |
| UNTRUSTWORTHY | NEGATIVE | 0 |
| UNKIND | NEGATIVE | -0.3818 |
| INSINCERE | NEGATIVE | -0.4215 |
| GREEDY | NEGATIVE | -0.3182 |
| CONCEITED | NEGATIVE | 0 |
| RUDE | NEGATIVE | -0.4588 |
| HOSTILE | NEGATIVE | -0.3818 |
| UNFRIENDLY | NEGATIVE | -0.3612 |
| NOUN | NEUTRAL | 0 |
| ADJECTIVE | NEUTRAL | 0 |
| VERB | NEUTRAL | 0 |
| WORD | NEUTRAL | 0 |
| MAGAZINE | NEUTRAL | 0 |
| BOOK | NEUTRAL | 0 |
| NEWSPAPER | NEUTRAL | 0 |
| PAMPHLET | NEUTRAL | 0 |
| PAPER | NEUTRAL | 0 |
| COTTON | NEUTRAL | 0 |
| WOOL | NEUTRAL | 0 |
| NYLON | NEUTRAL | 0 |
| CHAIR | NEUTRAL | 0 |
| TABLE | NEUTRAL | 0 |
| SOFA | NEUTRAL | 0 |
| DESK | NEUTRAL | 0 |

# 2. Supplementary Results

## 2.1. Participant Demographics

| 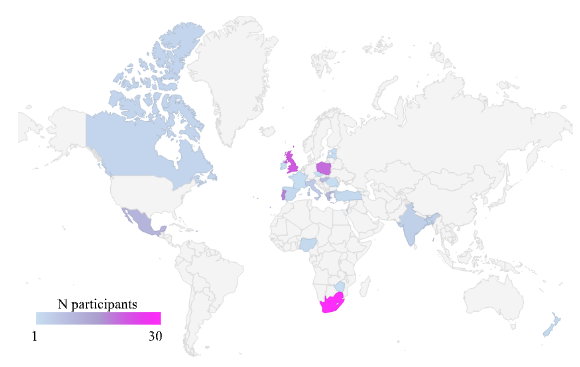 | **Nationality** | **N** |
| --- | --- | --- |
|  | South Africa  UK  Poland  Portugal  Mexico  Greece  Hungary  Italy  India  Canada  New Zealand  Nigeria  Puerto Rico  Spain  Turkey  Czech Republic  Estonia  France  Ireland  Israel  Latvia  Lesotho  Luxembourg  Romania  Zimbabwe  Bangladesh | 30  22  20  18  10  9  9  5  4  3  2  2  2  2  2  1  1  1  1  1  1  1  1  1  1  1 |

*Figure 1: Participant nationality world map.*

*Table 2: Frequency of first languages spoken by participants*

| **Language** | ***n*** | **%** |
| --- | --- | --- |
| Afrikaans | 2 | 1.3 |
| Bengali | 1 | 0.7 |
| Catalan | 1 | 0.7 |
| Czech | 1 | 0.7 |
| English | 61 | 40.4 |
| Estonian | 1 | 0.7 |
| German | 1 | 0.7 |
| Greek | 9 | 6.0 |
| Hindi | 1 | 0.7 |
| Hungarian | 8 | 5.3 |
| Italian | 5 | 3.3 |
| Latvian | 1 | 0.7 |
| Other | 2 | 1.3 |
| Polish | 21 | 13.9 |
| Portuguese | 17 | 11.3 |
| Romanian | 2 | 1.3 |
| Russian | 1 | 0.7 |
| Spanish | 12 | 7.9 |
| Tamil | 1 | 0.7 |
| Telugu | 1 | 0.7 |
| Turkish | 2 | 1.3 |

## 2.2. Experimental Stroop results

*Table 3: Regression table for generalized linear mixed effects model for standard Stroop experiment. To correct for multiple comparisons, we take a statistical significance threshold as 𝛼=0.025.*

|  | **rt** | | |
| --- | --- | --- | --- |
| *Predictors* | *Estimates* | *CI* | *p* |
| (Intercept) | 600.24 | 310.06 – 890.41 | **<0.001** |
| counterbalance | -125.92 | -306.06 – 54.23 | 0.171 |
| congruency [incongruent] | 89.69 | 30.45 – 148.92 | **0.003** |
| brooding | 1.83 | -22.00 – 25.65 | 0.881 |
| sex | 30.42 | -31.02 – 91.86 | 0.332 |
| age | 6.17 | 3.45 – 8.89 | **<0.001** |
| counterbalance ×  congruency [incongruent] | 38.12 | -0.99 – 77.23 | 0.056 |
| counterbalance × brooding | 8.03 | -7.89 – 23.95 | 0.323 |
| congruency [incongruent]  × brooding | 7.47 | 2.31 – 12.62 | **0.005** |
| (counterbalance ×  congruency [incongruent])  × brooding | -4.27 | -7.71 – -0.82 | **0.015** |
| **Random Effects** | | | |
| σ^2^ | 82046.56 | | |
| τ_00_ _pid_ | 35108.19 | | |
| ICC | 0.30 | | |
| N _pid_ | 151 | | |
| Observations | 28916 | | |
| Marginal R^2^ / Conditional R^2^ | 0.102 / 0.371 | | |

*Table 4: Regression table for generalized linear mixed effects model for emotional Stroop experiment. To correct for multiple comparisons, we take a statistical significance threshold as 𝛼=0.025.*

|  | **rt** | | |
| --- | --- | --- | --- |
| *Predictors* | *Estimates* | *CI* | *p* |
| (Intercept) | 439.69 | 169.02 – 710.36 | **0.001** |
| counterbalance | -17.10 | -187.53 – 153.33 | 0.844 |
| valence_score | -4.38 | -94.91 – 86.14 | 0.924 |
| brooding | 8.73 | -13.82 – 31.27 | 0.448 |
| sex | 36.08 | -22.39 – 94.55 | 0.226 |
| age | 6.40 | 3.82 – 8.99 | **<0.001** |
| counterbalance × valence_score | 16.72 | -43.07 – 76.52 | 0.584 |
| counterbalance × brooding | 5.75 | -9.31 – 20.81 | 0.454 |
| scores × brooding | -1.39 | -9.24 – 6.47 | 0.729 |
| (counterbalance × valence_score)  × brooding | 1.35 | -3.91 – 6.61 | 0.614 |
| **Random Effects** | | | |
| σ^2^ | 72037.18 | | |
| τ_00_ _pid_ | 31805.18 | | |
| ICC | 0.31 | | |
| N _pid_ | 151 | | |
| Observations | 28917 | | |
| Marginal R^2^ / Conditional R^2^ | 0.075 / 0.358 | | |

*Table 5: Regression table for generalized linear mixed effects model for standard Stroop experiment, with random slope for congruency. To correct for multiple comparisons, we take a statistical significance threshold as 𝛼=0.025.*

|  | **rt** | | |
| --- | --- | --- | --- |
| *Predictors* | *Estimates* | *CI* | *p* |
| (Intercept) | 599.76 | 320.99 – 878.53 | **<0.001** |
| counterbalance | -128.04 | -300.99 – 44.91 | 0.147 |
| congruency [incongruent] | 89.59 | -28.70 – 207.89 | 0.138 |
| brooding | 1.26 | -21.61 – 24.14 | 0.914 |
| sex | 38.14 | -21.16 – 97.43 | 0.207 |
| age | 6.01 | 3.38 – 8.63 | **<0.001** |
| counterbalance ×  congruency [incongruent] | 38.23 | -39.93 – 116.39 | 0.338 |
| counterbalance × brooding | 8.24 | -7.04 – 23.53 | 0.290 |
| congruency [incongruent]  × brooding | 7.48 | -2.81 – 17.76 | 0.154 |
| (counterbalance ×  congruency [incongruent])  × brooding | -4.28 | -11.17 – 2.61 | 0.224 |
| **Random Effects** | | | |
| σ^2^ | 80785.14 | | |
| τ_00_ _pid_ | 32301.92 | | |
| τ_11_ _pid.congruencyincongruent_ | 5165.44 | | |
| ρ_01_ _pid_ | 0.11 | | |
| ICC | 0.31 | | |
| N _pid_ | 151 | | |
| Observations | 28916 | | |
| Marginal R^2^ / Conditional R^2^ | 0.102 / 0.380 | | |

*Table 6: Regression table for generalized linear mixed effects model for emotional Stroop experiment, with confounding stimuli (“GREEDY” and “RUDE”) excluded.*

|  | **rt** | | |
| --- | --- | --- | --- |
| Predictors | Estimates | CI | p |
| (Intercept) | 442.15 | 170.67 – 713.62 | **0.001** |
| counterbalance | -17.26 | -188.19 – 153.68 | 0.843 |
| scores | -8.53 | -103.55 – 86.50 | 0.860 |
| rrs | 8.50 | -14.11 – 31.11 | 0.461 |
| sex s | 37.54 | -21.10 – 96.18 | 0.210 |
| age | 6.38 | 3.78 – 8.97 | **<0.001** |
| counterbalance × scores | 15.43 | -47.29 – 78.14 | 0.630 |
| counterbalance × rrs | 5.79 | -9.31 – 20.90 | 0.452 |
| scores × rrs | -0.90 | -9.13 – 7.33 | 0.830 |
| (counterbalance × scores)  × rrs | 1.35 | -4.16 – 6.86 | 0.630 |
| **Random Effects** | | | |
| σ^2^ | 72595.17 | | |
| τ_00_ _pid_ | 31971.05 | | |
| ICC | 0.31 | | |
| N _pid_ | 151 | | |
| Observations | 27650 | | |
| Marginal R^2^ / Conditional R^2^ | 0.074 / 0.357 | | |

*Table 7: Regression table for generalized linear mixed effects model for emotional Stroop experiment, with english as a second language (ESL) included as a covariate, and confounding stimuli (“GREEDY” and “RUDE”) excluded.*

|  | **rt** | | |
| --- | --- | --- | --- |
| *Predictors* | *Estimates* | *CI* | *p* |
| (Intercept) | 483.84 | 213.29 – 754.39 | **<0.001** |
| counterbalance | -0.78 | -170.15 – 168.59 | 0.993 |
| scores | -8.53 | -103.56 – 86.49 | 0.860 |
| rrs | 9.04 | -13.28 – 31.37 | 0.427 |
| sex s | 39.14 | -18.76 – 97.04 | 0.185 |
| age | 5.85 | 3.24 – 8.45 | **<0.001** |
| ESL [NonNativeEng] | -66.18 | -125.30 – -7.06 | **0.028** |
| counterbalance × scores | 15.43 | -47.29 – 78.14 | 0.630 |
| counterbalance × rrs | 4.77 | -10.17 – 19.71 | 0.532 |
| scores × rrs | -0.90 | -9.13 – 7.33 | 0.829 |
| (counterbalance × scores)  × rrs | 1.35 | -4.16 – 6.86 | 0.630 |
| **Random Effects** | | | |
| σ^2^ | 72595.17 | | |
| τ_00_ _pid_ | 31141.44 | | |
| ICC | 0.30 | | |
| N _pid_ | 151 | | |
| Observations | 27650 | | |
| Marginal R^2^ / Conditional R^2^ | 0.083 / 0.358 | | |

### 2.2.1. Power analyses for random slopes model

We conducted simulation-based power analyses using the *simr* package in R to estimate the sample size required to detect the three-way interaction under mixed models with 1) a random intercept for participants only (1 | PID), and 2) a model that also includes a random slope to capture individual variability in congruency effect (1 + congruency | PID). For the models with a random intercept only, we assumed a between-subject baseline variability in reaction time as 400ms, and a residual variance of 250ms. For the models including a random slope, we constructed the covariance matrix by assuming within-subject congruency variability of 100ms, and correlation of 0.7 between baseline reaction time variability and congruency effect. For this more complex model, we used a residual variability of 50ms. To detect the three-way (*counterbalancing x congruency x brooding*) interaction at an effect size of 0.05 (corresponding to ~20ms), the random intercept model requires at least 100 participants, while the random slope model requires ~1100 (Figure 2), taking α = 0.05. Indeed, a sample size of 150, as included in the present study, yields an insufficient power of 16.8% (95% confidence interval: 13.63–20.37) for the three-way interaction taking α = 0.025. Therefore, the present study is underpowered for a statistical model including a random slope.


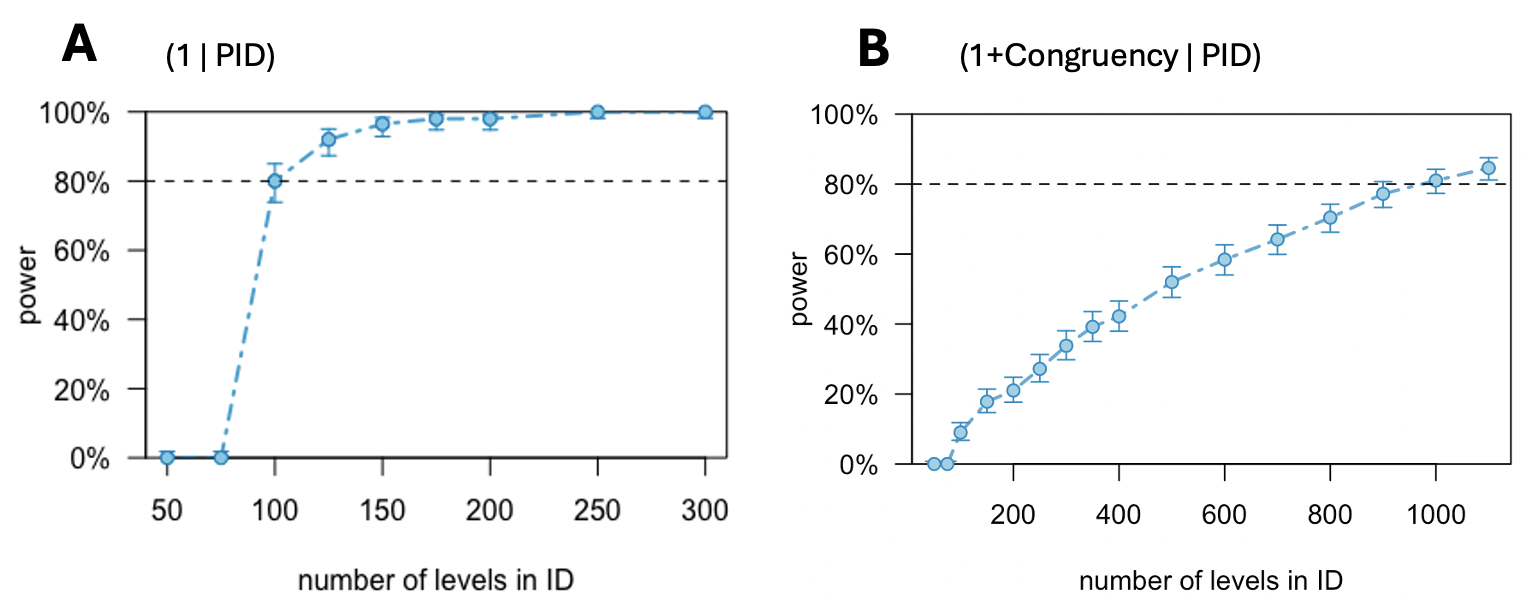


*Figure 2: Power curves for mixed models with A) random intercept for participants, and B) random slope for congruency by participant, for the standard Stroop data only.*

## 2.3. Computational Modelling Results


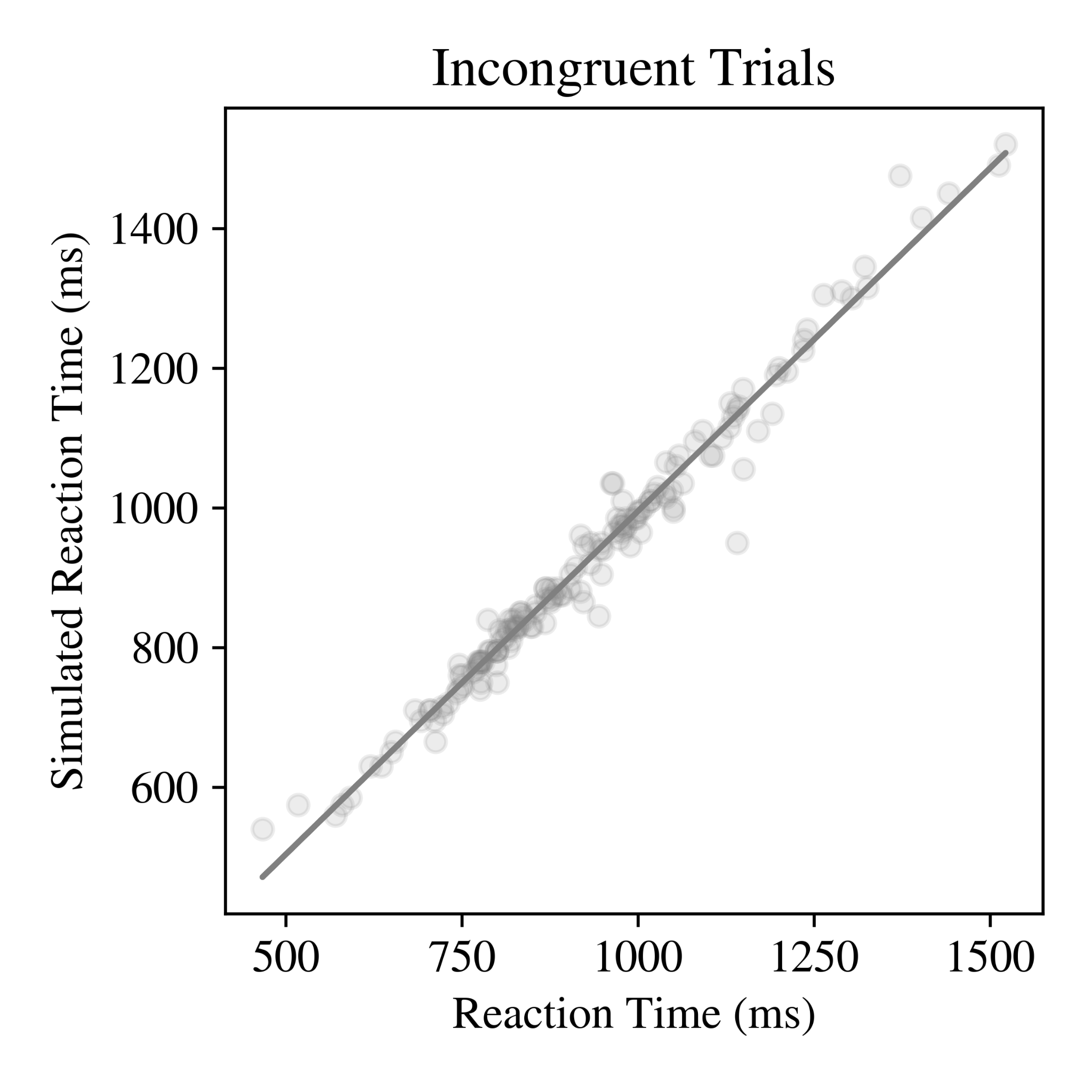

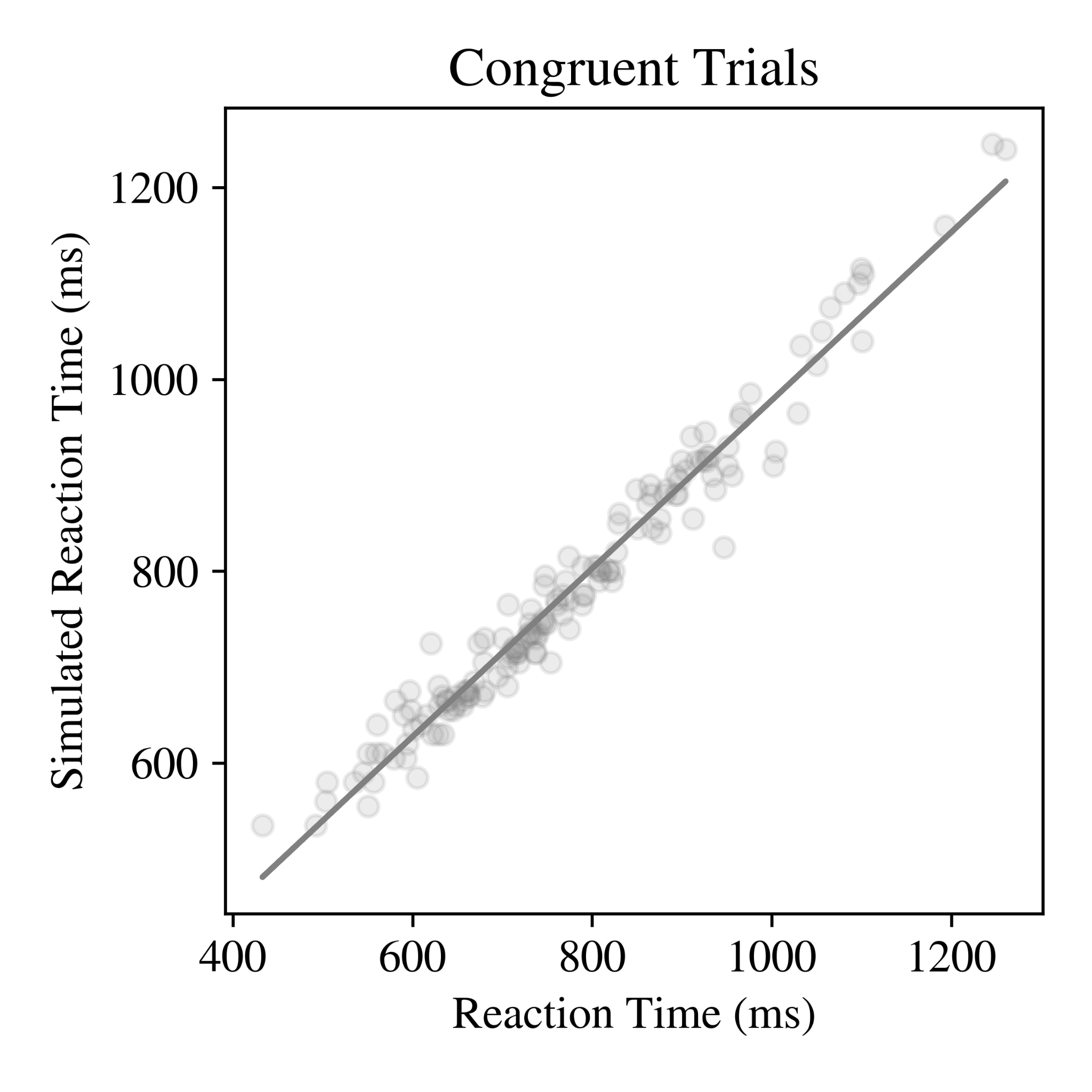


**A**

**B**

*Figure 3: Our numerical parameter fitting procedure yielded model results that align with experimental reaction times for both A) incongruent trials, and B) congruent trials of the standard Stroop task. Results are shown for the selected “full” model. Linear modelling confirmed that these relationships are significant (congruent trials: R^2^=0.965, p<0.001; incongruent trials: R^2^=0.976, p<0.001)*.

*Table 8: Regression table for computational modelling parameters for standard Stroop task.*

|  | **z-rrsbrooding** | | |
| --- | --- | --- | --- |
| *Predictors* | *Estimates* | *CI* | *p* |
| (Intercept) | -0.60 | -1.27 – 0.07 | 0.080 |
| emotion task w | 0.19 | 0.04 – 0.34 | **0.015** |
| task emotion w | 0.14 | -0.02 – 0.29 | 0.092 |
| cb | 0.06 | -0.25 – 0.37 | 0.684 |
| task intg | -0.24 | -0.72 – 0.24 | 0.321 |
| emotion intg | -0.37 | -0.86 – 0.12 | 0.142 |
| task bias | 0.07 | -0.08 – 0.22 | 0.372 |
| task gain | 0.26 | 0.11 – 0.41 | **0.001** |
| emotion bias | -0.07 | -0.22 – 0.08 | 0.327 |
| emotion gain | -0.03 | -0.18 – 0.12 | 0.669 |
| sex | 0.32 | 0.03 – 0.61 | **0.029** |
| age | -0.29 | -0.44 – -0.14 | **<0.001** |
| task intg × emotion intg | 0.64 | 0.21 – 1.07 | **0.004** |
| cb × task intg | 0.18 | -0.12 – 0.49 | 0.236 |
| cb × emotion intg | 0.25 | -0.06 – 0.56 | 0.113 |
| (cb × task intg) ×  emotion intg | -0.47 | -0.76 – -0.19 | **0.001** |
| Observations | 151 | | |
| R^2^ / R^2^ adjusted | 0.310 / 0.234 | | |

*Table 9: Regression table for* post hoc *analysis of three-way interaction (cb x task_intg x emotion_intg): first counterbalancing condition.*

|  | **z-rrsbrooding** | | |
| --- | --- | --- | --- |
| Predictors | Estimates | CI | p |
| (Intercept) | -0.05 | -0.29 – 0.19 | 0.677 |
| task intg | -0.02 | -0.26 – 0.23 | 0.896 |
| emotion intg | -0.07 | -0.32 – 0.18 | 0.575 |
| task intg × emotion intg | 0.16 | -0.05 – 0.38 | 0.129 |
| Observations | 76 | | |
| R^2^ / R^2^ adjusted | 0.045 / 0.005 | | |

*Table 10: Regression table for* post hoc *analysis of three-way interaction (cb x task_intg x emotion_intg): second counterbalancing condition.*

|  | **z-rrsbrooding** | | |
| --- | --- | --- | --- |
| *Predictors* | *Estimates* | *CI* | *p* |
| (Intercept) | 0.01 | -0.21 – 0.23 | 0.919 |
| task intg | 0.08 | -0.14 – 0.30 | 0.471 |
| emotion intg | 0.12 | -0.10 – 0.34 | 0.290 |
| task intg × emotion intg | -0.33 | -0.56 – -0.10 | **0.005** |
| Observations | 75 | | |
| R^2^ / R^2^ adjusted | 0.118 / 0.081 | | |


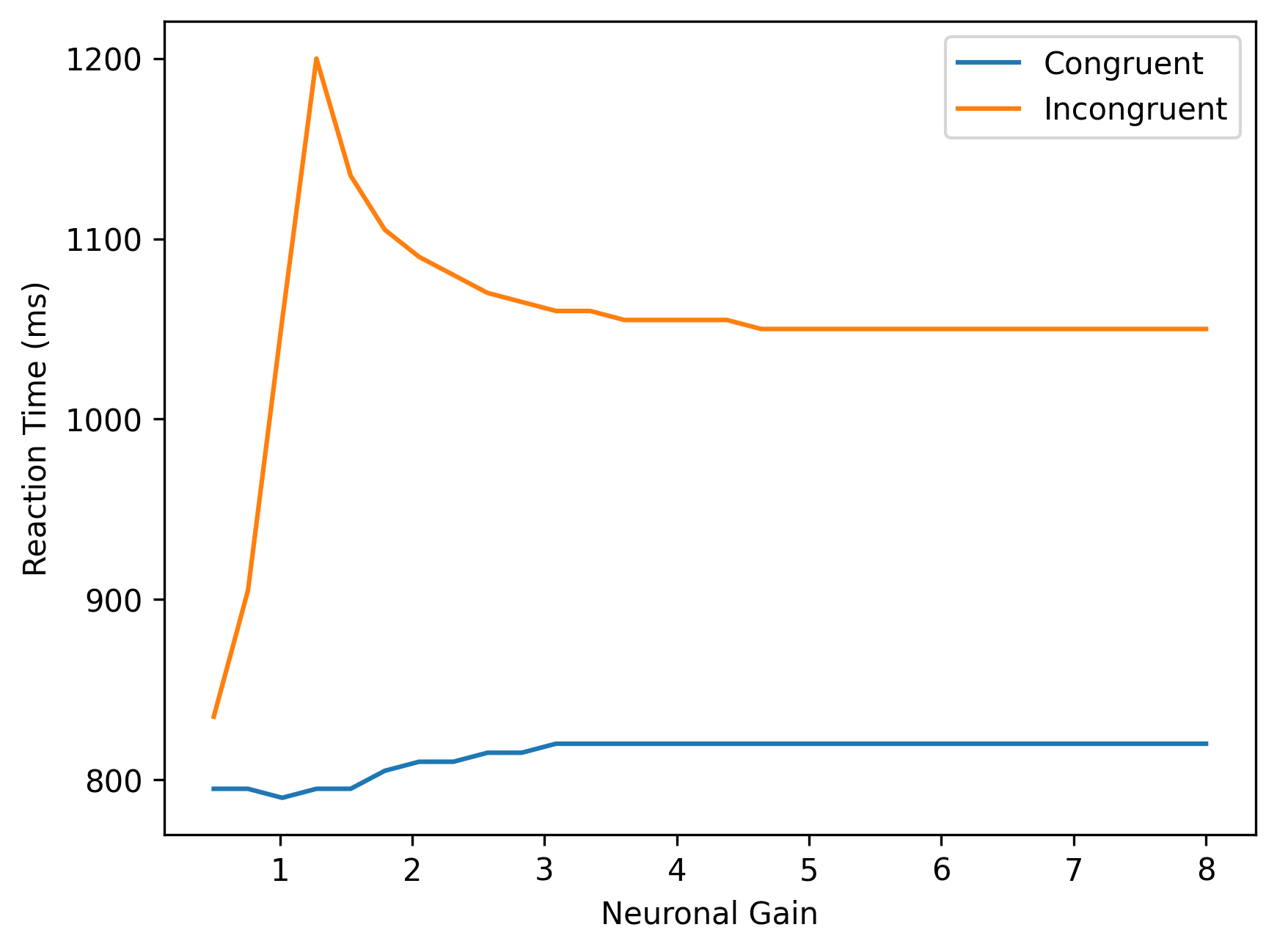


*Figure 4: Stroop interference effect in relation to neuronal gain changes in the task control layer. Increases in neuronal gain, especially in smaller values, enhance the Stroop interference effect in our computational model. Increasing neuronal gain may make the neurons more sensitive to changes in input, which in this case contributes to poor cognitive flexibility, reflected through the exacerbated Stroop interference effect.*

## 2.4. Model selection procedure


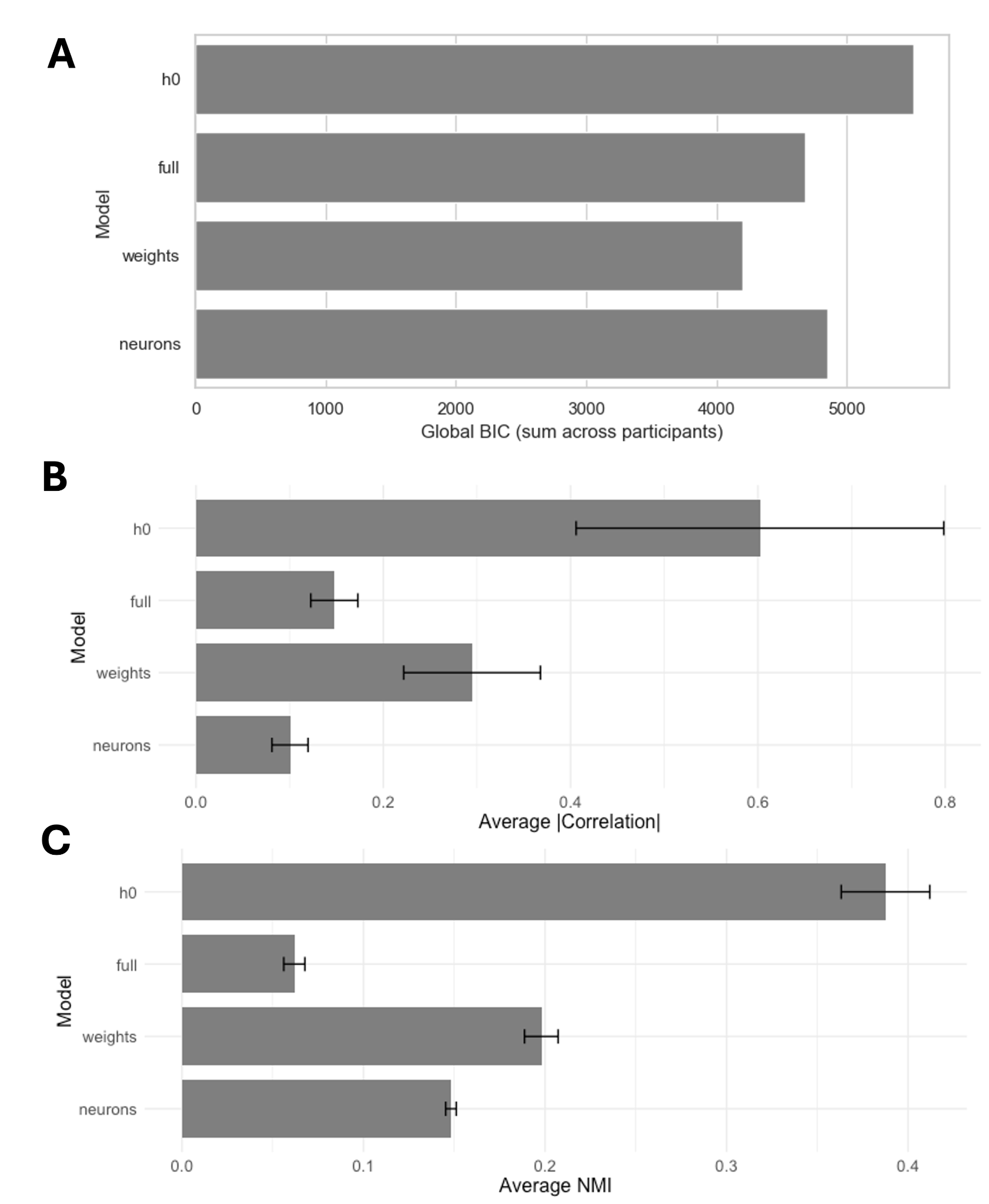


*Figure 5: A) Global BIC scores, B) average absolute pairwise correlation between parameters, and C) average normalized mutual information (NMI), for the four compared models. Error bars show standard error of the mean. Higher averaged absolute correlations and normalized mutual information indicate higher levels of parameter interdependence; therefore, to maximize interpretability of parameters, a model that has a low value in these indices is preferred. The four models are: 1) a base model in which only the GRAIN model weights were fitted (h0); 2) a weights model, which added the task control↔emotion layer weights to those in the base model; 3) a neuron model, which added biases, gains, and integration rates within the task control and emotion hidden layers to the base model weights; and 4) a full model, which included all parameters weights and neuron-level parameters. While the ‘weights’ model demonstrates the lowest BIC, it also has high averaged absolute correlations and normalized mutual information between parameters. The ‘full’ model demonstrates the second lowest BIC, second lowest averaged absolute Pearson correlation, and lowest normalized mutual information, which is why it was selected for subsequent analyses and interpretation.*


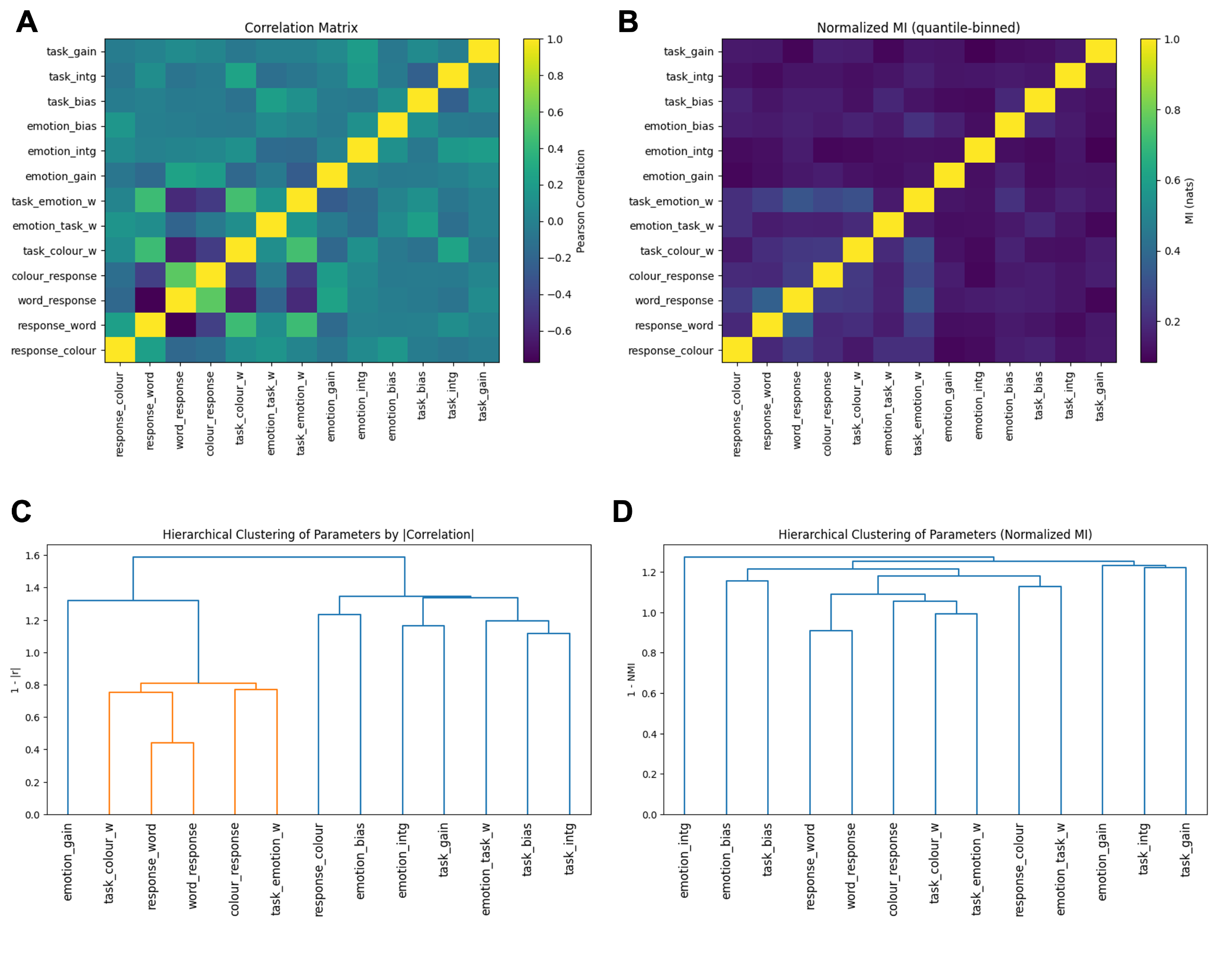


*Figure 6: Parameter correlations (A), normalized mutual information (NMI) (B) and dendrograms (C-D) for the “full” model. NMI reveals nonlinear dependencies that may be missed by using Pearson correlations alone; this is highly relevant when investigating relationships between neuron-level parameters. Hierarchical clustering may be useful for informing future reparameterization strategies by revealing clusters of parameters that are highly related, and thus should be numerically “tied” together or represented by a single parameter. Model weights (“task_colour_w”, “response_word”, “word_response”, “colour_response”, “task_emotion_w”) cluster together in both dendrograms, suggesting strong dependencies between these parameters. The NMI dendrogram also demonstrates clusters of neuron-level parameters (e.g., “emotion_gain”, “task_intg”, “task_gain”), suggesting nonlinear dependencies.*

## 2.5. Parameter Recovery


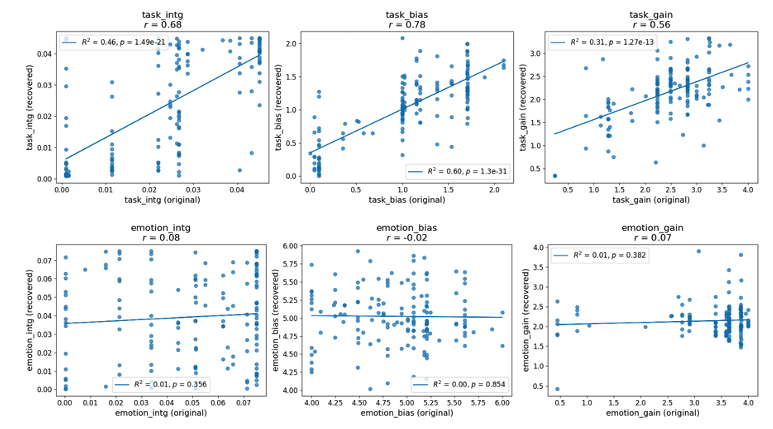


*Figure 7: Parameter recovery for neuron-level parameters after fixing model weights. Parameters for the task layer were fairly-moderately recoverable, whereas the parameters for the emotion layer were unrecoverable.*

*
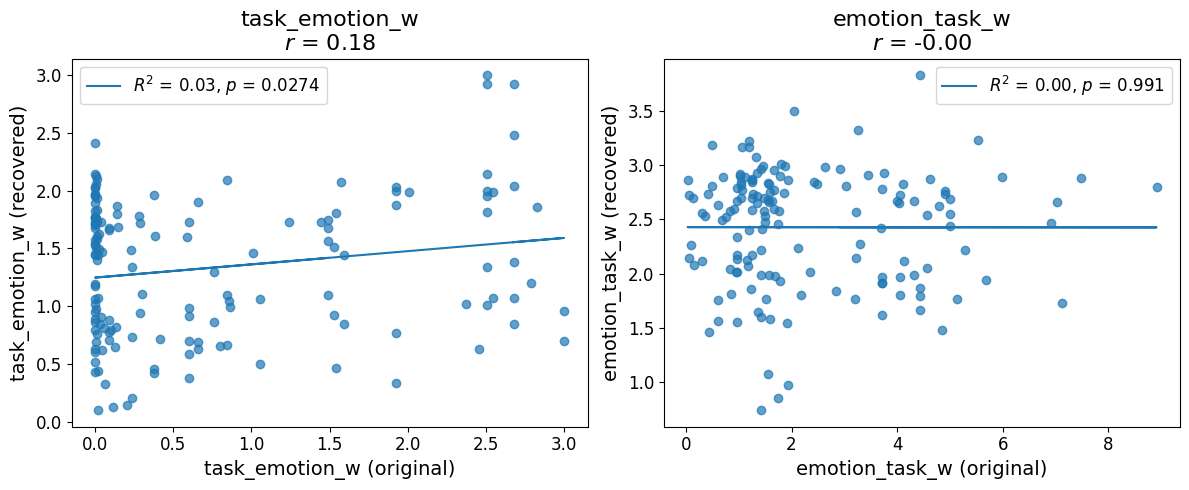
*

*Figure 8: Parameter recovery for task→emotion and emotion→task weights after fixing neuron-level parameters. Weights were unrecoverable.*
